# The role of the MAD2-TLR4-MyD88 axis in paclitaxel resistance in ovarian cancer

**DOI:** 10.1371/journal.pone.0243715

**Published:** 2020-12-28

**Authors:** Mark Bates, Cathy D. Spillane, Michael F. Gallagher, Amanda McCann, Cara Martin, Gordon Blackshields, Helen Keegan, Luke Gubbins, Robert Brooks, Doug Brooks, Stavros Selemidis, Sharon O’Toole, John J. O’Leary

**Affiliations:** 1 Department of Histopathology, Trinity College Dublin, Dublin, Ireland; 2 Emer Casey Molecular Pathology Research Laboratory, Coombe Women & Infants University Hospital, Dublin, Ireland; 3 Trinity St James’s Cancer Institute, Dublin, Ireland; 4 Department of Obstetrics and Gynaecology, Trinity College Dublin, Dublin, Ireland; 5 College of Health Sciences, University College Dublin, Belfield, Dublin, Ireland; 6 Department of Pathology, Coombe Women & Infants University Hospital, Dublin, Ireland; 7 School of Pharmacy and Medical Sciences, University of South Australia, Adelaide, Australia; 8 School of Health and Biomedical Sciences, Royal Melbourne Institute of Technology, Bundoora, Australia; The University of Hong Kong, HONG KONG

## Abstract

Despite the use of front-line anticancer drugs such as paclitaxel for ovarian cancer treatment, mortality rates have remained almost unchanged for the past three decades and the majority of patients will develop recurrent chemoresistant disease which remains largely untreatable. Overcoming chemoresistance or preventing its onset in the first instance remains one of the major challenges for ovarian cancer research. In this study, we demonstrate a key link between senescence and inflammation and how this complex network involving the biomarkers MAD2, TLR4 and MyD88 drives paclitaxel resistance in ovarian cancer. This was investigated using siRNA knockdown of MAD2, TLR4 and MyD88 in two ovarian cancer cell lines, A2780 and SKOV-3 cells and overexpression of MyD88 in A2780 cells. Interestingly, siRNA knockdown of MAD2 led to a significant increase in TLR4 gene expression, this was coupled with the development of a highly paclitaxel-resistant cell phenotype. Additionally, siRNA knockdown of MAD2 or TLR4 in the serous ovarian cell model OVCAR-3 resulted in a significant increase in TLR4 or MAD2 expression respectively. Microarray analysis of SKOV-3 cells following knockdown of TLR4 or MAD2 highlighted a number of significantly altered biological processes including EMT, complement, coagulation, proliferation and survival, ECM remodelling, olfactory receptor signalling, ErbB signalling, DNA packaging, Insulin-like growth factor signalling, ion transport and alteration of components of the cytoskeleton. Cross comparison of the microarray data sets identified 7 overlapping genes including MMP13, ACTBL2, AMTN, PLXDC2, LYZL1, CCBE1 and CKS2. These results demonstrate an important link between these biomarkers, which to our knowledge has never before been shown in ovarian cancer. In the future, we hope that triaging patients into alterative treatment groups based on the expression of these three biomarkers or therapeutic targeting of the mechanisms they are involved in will lead to improvements in patient outcome and prevent the development of chemoresistance.

## Introduction

Ovarian cancer is a major cause of cancer death in women worldwide with less than 40% of women surviving beyond 5 years post-diagnosis [1]. This is due mainly to the development of recurrent chemoresistant disease which cannot as of yet be effectively treated in patients once it develops [2]. To improve patient outcomes, we must be able to either effectively destroy chemoresistant tumours once they resurface or prevent them from developing in the first instance. One way in which their development could be prevented is through the use of prognostic biomarkers which can identify patients who will likely develop chemoresistance prior to the commencement of treatment. These patients once identified could be selected out from the main patient population and given more appropriate treatments to prevent the onset of chemoresistance. In recent years our group and others have extensively investigated three new prognostic biomarkers, known as toll-like receptor 4 (TLR4), myeloid differentiation factor 88 (MyD88) and mitotic arrest deficient 2 (MAD2) for the most common and lethal form of ovarian cancer; high grade serous ovarian cancer (HGSOC). All three markers have been shown to be involved in the development of chemoresistance to paclitaxel [3–10], one of the first-line chemotherapies used to treat ovarian cancer and their expression levels have been shown to correlate with poor clinical outcome in patients [6, 7, 11–16]. TLR4 is an innate immune receptor responsible for the recognition of lipopolysaccharide (LPS) on gram-negative bacteria. Upon ligand engagement, TLR4 activates inflammatory cytokine production through its downstream adaptor molecule MyD88. Activation of this signalling pathway is thought to drive tumour-associated inflammation, resistance to apoptosis and promote the induction of a stem-like phenotype [3, 17–19]. Paclitaxel, due to its homology to LPS [20] and its ability to bind TLR4 and activate downstream signalling, is thought to promote the development of this aggressive phenotype [17, 18, 21, 22]. Elevated expression levels of TLR4 or it’s adaptor protein MyD88 have been associated with reduced survival outcome in HGSOC patients [6, 8, 15, 23–25], while therapeutic targeting of TLR4 has been shown to restore paclitaxel sensitivity in ovarian cancer cell models [6, 16]. Although, it must be acknowledged that a recent largescale study found no prognostic association between TLR4 expression and HGSOC [24], despite previous contra-indications in a smaller study by the same group, particularly when TLR4 was combined with MyD88 [23]. Interestingly however the TLR4 downstream adaptor molecule MyD88 was found to be prognostic in this largescale cohort in agreement with a number of other studies including our own [6, 8, 15, 23–25]. Given these findings and the fact that paclitaxel is a known ligand for TLR4 [20], further interrogation of how this pathway contributes to paclitaxel chemoresistance is warranted.

MAD2 is a key component of the spindle assembly checkpoint (SAC) responsible for correct segregation of chromosomes during cell division. Suppression of MAD2 leads to mitotic catastrophe as cells divide without proper chromosomal segregation. This leads to anaphase bridge formation and generation of a DNA damage response which mimics normal telomere shortening resulting in the induction of cellular senescence [5, 26]. Cellular senescence allows tumour cells to resist paclitaxel which only targets actively dividing cells while also promoting tumour growth through the release of a milieu of over 40 different cytokines/chemokines and other factors as part of what is known as the senescence associated secretory phenotype (SASP) [27]. As all three biomarkers have been shown, individually, to have a significant impact on patient prognosis and the modulation of paclitaxel chemoresponsiveness and also given the fact that many cytokines secreted during senescence are also known downstream targets of TLR4-MyD88 signalling we hypothesised that there may be crosstalk between these three important biomarkers in ovarian cancer. The aim of this study, therefore, was to assess whether there was any molecular link between MAD2, TLR4 and MyD88 in ovarian cancer and to further explore the mechanisms each of these biomarkers utilise, in order to render ovarian cancer cells resistant to paclitaxel therapy.

## Results

### Identifying the molecular link between MAD2 and TLR4-MyD88 signalling

**Fig 1:**
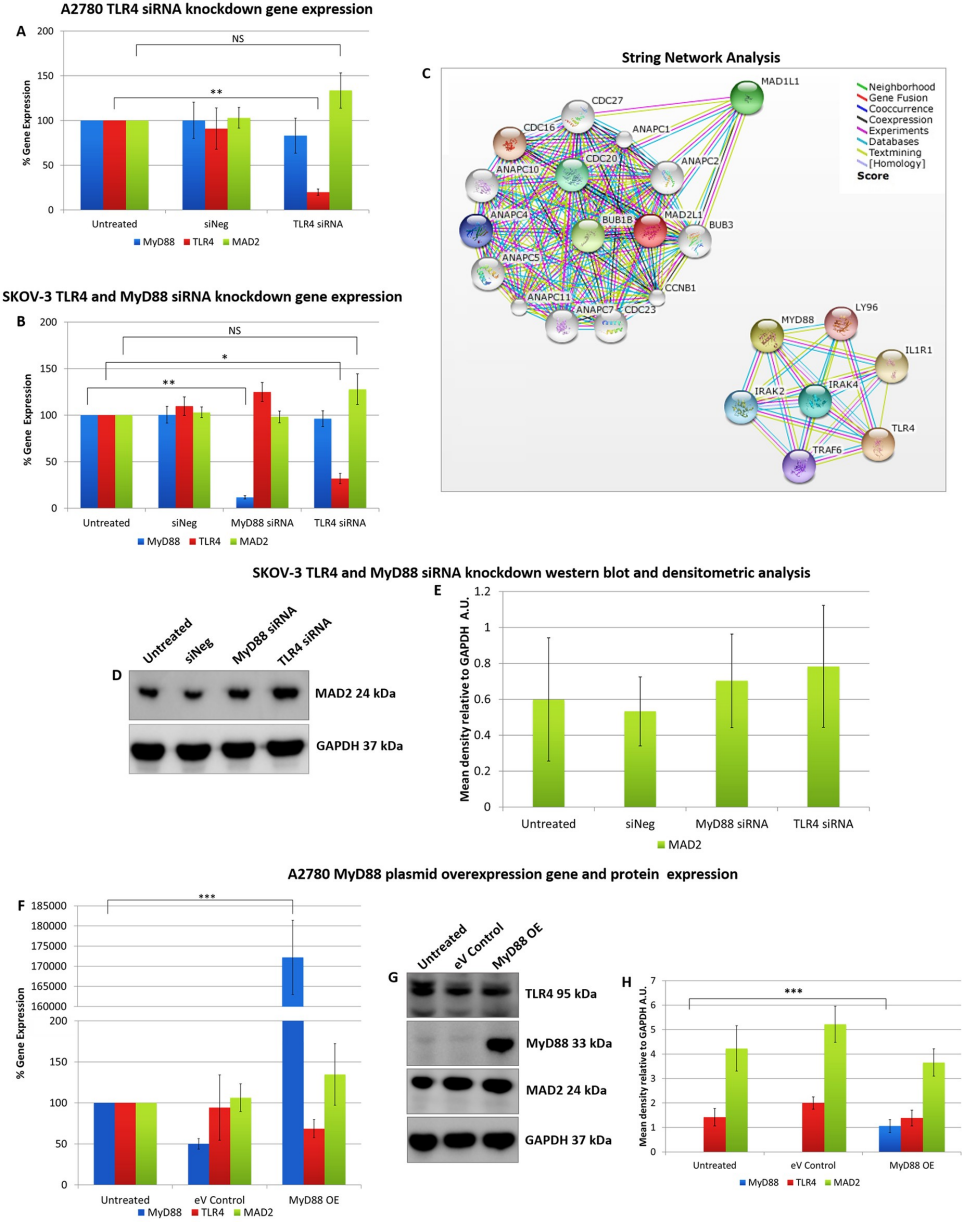
Alteration of TLR4 and MyD88 expression does not alter MAD2 expression in A2780 or SKOV-3 cells. MyD88, TLR4 and MAD2 gene expression levels in (A) A2780 and (B) SKOV-3 cells, 72 hours following transfection with siRNA targeting TLR4 or MyD88. Neither knockdown of TLR4 in both SKOV-3 and A2780 cells or knockdown of MyD88 in SKOV-3 cells had any significant impact on the expression of MAD2. (C) Screenshots from the STRING website, which was queried for relationships between TLR4, MyD88 and MAD2. Coloured lines between the proteins indicate the various types of interaction evidence. *In-silico* analysis predicted that there was no direct interaction between MAD2 and MyD88 or TLR4. (D) Western blot analysis and (E) densitometric analysis of MAD2 protein expression levels in SKOV-3 cells following transfection with siRNA targeting MyD88 or TLR4. (F) MyD88, TLR4 and MAD2 gene expression levels, (G) western blot analysis and (H) densitometric analysis of A2780 cells transfected with a MyD88 overexpression plasmid for 72 hours. The results demonstrate that overexpression of MyD88 had no significant impact on MAD2 gene or protein expression. Results are expressed as mean +/-SD, at least n = 3; NS—Not significant, *p<0.05, **p<0.01, ***p<0.01 (Student’s t-test). Densitometry results are expressed in arbitrary units (A.U) normalised to GAPDH. **Note**:- Blots are cropped from original images available in the S1 Raw Images.

In order to discern a possible relationship between MAD2 and TLR4-MyD88 signalling, transfection experiments were performed initially in both A2780 (MyD88 null) and SKOV-3 (MyD88 positive) ovarian cancer cells (Fig 1). Firstly, TLR4 was knocked down in both cell models using siRNA. Secondly MyD88 was knocked down in SKOV-3 cells while A2780 cells were transfected with a MyD88 overexpression plasmid. Following each transfection experiment MAD2 expression levels were assessed. Knockdown of TLR4 in both cell models did not alter MAD2 expression levels nor did knockdown or overexpression of MyD88 in SKOV-3 or A2780 cell lines respectively. Thus, indicating that TLR4-MyD88 signalling and MAD2 were independent or at the very least that MAD2 expression was not influenced by changes in TLR4 or MyD88 expression in these cell models. In parallel with this work, *in-silico* analysis was performed using the Search Tool for the Retrieval of Interacting Genes/Proteins (STRING) v10 software [28] in order to identify any potential interaction between the TLR4-MyD88 signalling pathway and MAD2. In support of the transfection experiments *in-silico* analysis identified no direct relationship between MAD2 and TLR4 or MyD88. TLR4, MyD88 and their interactants segregated into entirely different clusters than MAD2 and its interactants (Fig 1C).

**Fig 2:**
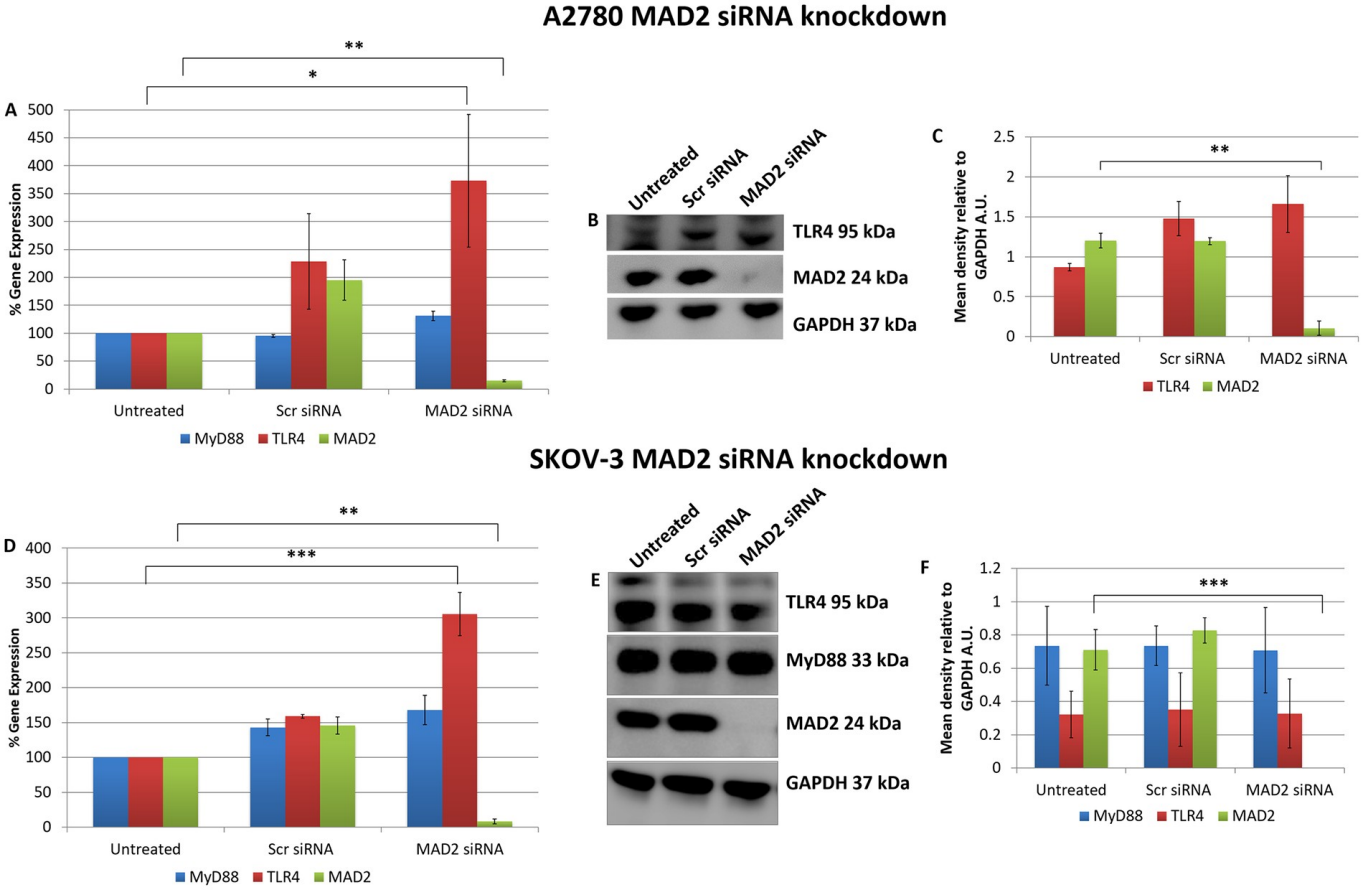
MAD2 is an inhibitor of TLR4 gene expression. MyD88, TLR4 and MAD2 gene expression levels in (A) A2780 and (D) SKOV-3 cells, 72 hours following transfection with siRNA targeting MAD2. Interestingly TLR4 gene expression, but not that of its adaptor molecule MyD88, was increased 3-fold following siRNA knockdown of MAD2 for 72 hours in both cell lines. (B) Western blot and (C) densitometry analysis of protein lysates harvested from A2780 cells following knockdown of MAD2 revealed however that suppression of MAD2 had no impact on TLR4 or MyD88 protein expression. Similarly, western blot (E) and densitometric analysis (F) in SKOV-3 cells found no significant increase in TLR4 protein expression post knockdown of MAD2. Results are expressed as mean +/-SD, n = 3; *p<0.05, **p<0.01, ***p<0.001 (Student’s t-test). Densitometry results are expressed in arbitrary units (A.U) normalised to GAPDH. **Note**:- Blots are cropped from original images available in the S1 Raw Images.

These initial transfection experiments and the *in-silico* analysis supported the idea that TLR4-MyD88 signalling and MAD2 acted as independent biomarkers in ovarian cancer. However, to conclusively demonstrate this, in the reverse setting, TLR4 and MyD88 expression was analysed following knockdown of MAD2 in both A2780 and SKOV-3 cells (Fig 2). Most interestingly when MAD2 levels were suppressed using siRNA, both A2780 and SKOV-3 cells exhibited a significant 3-fold increase in TLR4 gene expression, demonstrating a previously never-before shown link between TLR4 and MAD2 in ovarian cancer (Fig 2A and 2D). However, surprisingly a similar increase in TLR4 protein expression post knockdown of MAD2 was not observed at the selected timepoint in either cell line (Fig 2B and 2E).

**Fig 3:**
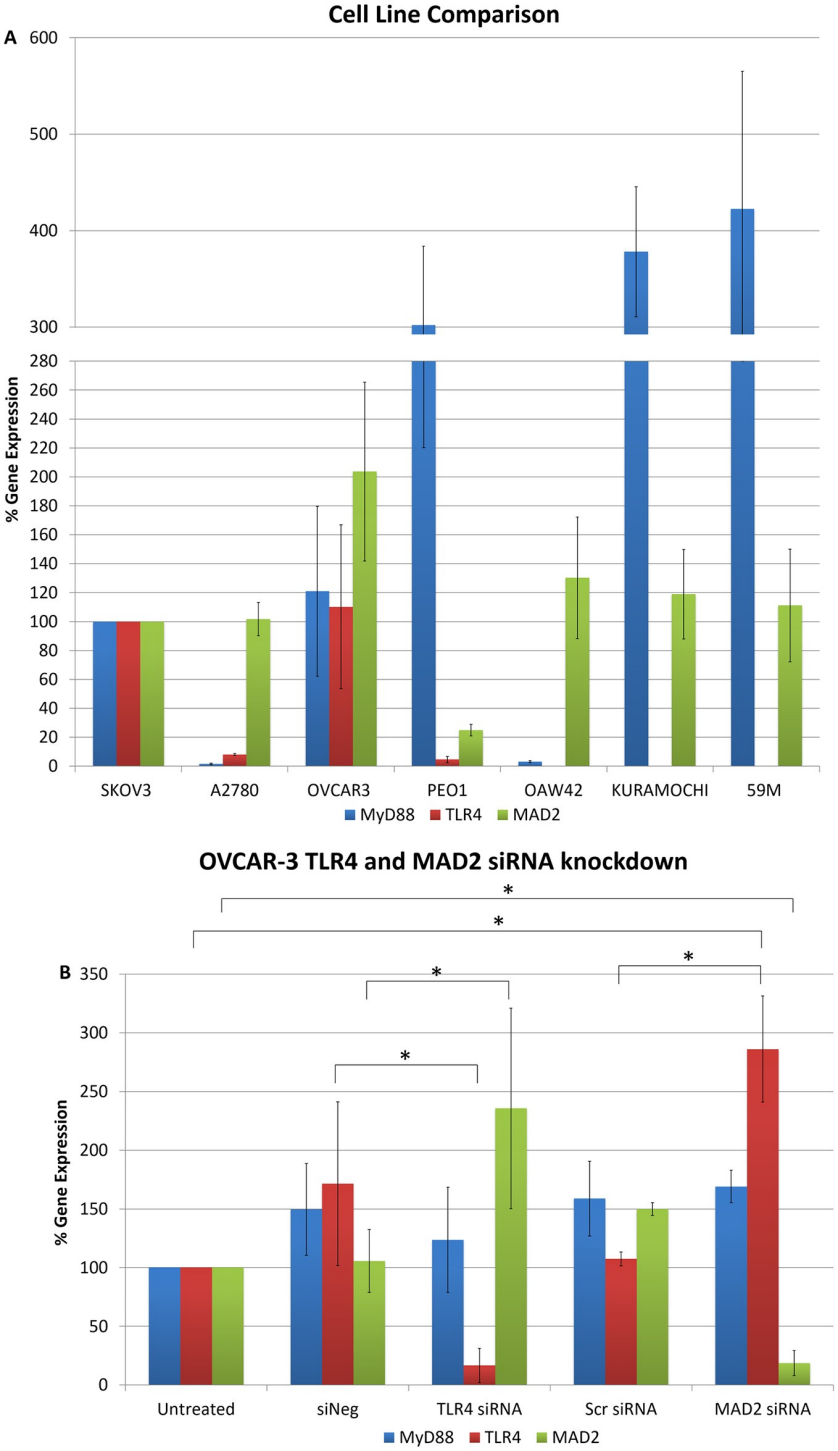
Cell line cross comparison and OVCAR-3 TLR4 and MAD2 siRNA knockdown. (A) MyD88, TLR4 and MAD2 gene expression in A2780, OVCAR-3, PEO1, OAW42, KURAMOCHI and 59M cells relative to SKOV-3 cells. (B) MyD88, TLR4 and MAD2 gene expression in OVCAR-3 cells following siRNA knockdown of TLR4 or MAD2. Gene expression levels were normalised to the endogenous control GAPDH and calibrated to that of untreated cells to establish the relative change in gene expression.

To further explore the link between TLR4 and MAD2 we next analysed the expression of MAD2, TLR4 and MyD88 in 5 additional ovarian cancer cell lines; OVCAR-3, PEO1, OAW42, KURAMOCHI and 59M cells (Fig 3A). Of these only OVCAR-3 and PEO1 expressed TLR4, MyD88 and MAD2. OAW42, KURAMOCHI and 59M were TLR4 negative. OVCAR-3 cells due to their TLR4 positivity and as a representative model of serous ovarian cancer were subsequently transfected with siRNA targeting TLR4 or MAD2 and then TLR4, MAD2 and MyD88 expression levels were assessed. Interestingly knockdown of TLR4 or MAD2 in the OVCAR-3 cell model caused a significant 2.4 and 2.9 fold increase in MAD2 or TLR4 expression respectively further highlighting an important link between these two biomarkers (Fig 3B).

### Suppression of MAD2 induces cellular senescence and paclitaxel resistance

**Fig 4:**
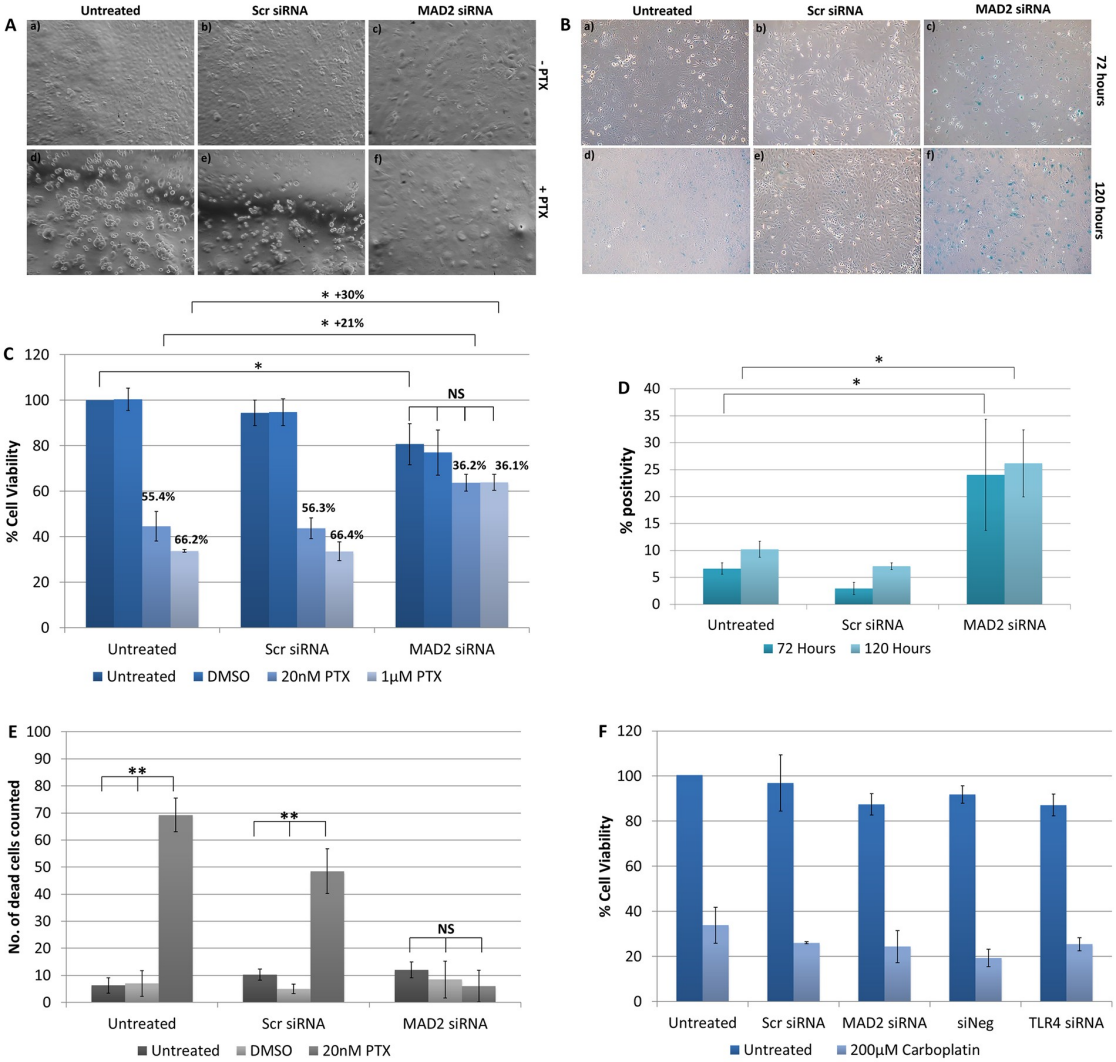
Suppression of MAD2 induces cellular senescence and paclitaxel resistance in SKOV-3 cells. (A) Representative images of untransfected SKOV-3 cells and SKOV-3 cells transfected with siRNA targeting MAD2 (MAD2 siRNA) or a scrambled negative control siRNA (Scr siRNA) for 72 hours. After 24 hours, cells were either left untreated (-PTX) (a-c) or were treated with a 1μM dose of paclitaxel (+PTX) (d-f) for a further 48 hours. (B) Representative images of SKOV-3 cells stained using the senescence β-galactosidase staining kit following transfection for 72 hours (a-c) or 120 hours (d-f). (C) CCK-8 assay results. % cell viability for each condition was calculated as a percentage of untransfected SKOV-3 cells which were left untreated. The results demonstrate that knockdown of MAD2 renders SKOV-3 cells resistant to paclitaxel. (D) The percentage of β-galactosidase positive cells was calculated for each condition for (n = 3) technical and (n = 3) biological replicates. Following transfection a 3-fold increase in β-galactosidase expression was observed demonstrating that knockdown of MAD2 induces cellular senescence in SKOV-3 cells. (E) Trypan blue exclusion assay. (F) CCK-8 assay results for SKOV-3 cells treated with 200μM carboplatin for 48 hours following a 24 hour transfection. Results are expressed as mean +/-SD, n = 3. *p<0.05, **p<0.01, ***p<0.001 (Student’s t-test).

Following knockdown of MAD2, SKOV-3 cells exhibited an increase in cell and nuclear size and alteration of cell shape (Fig 4A). Subsequently, the chemoresponse of SKOV-3 cells to paclitaxel following knockdown of MAD2 was assessed (Fig 4C). When SKOV-3 cells were treated with a 20nM or 1μM dose of paclitaxel following knockdown of MAD2, they exhibited a reduction in cell viability of 36.2% and 36.1% compared with untransfected cells which were not treated with paclitaxel. In contrast, untransfected cells or cells transfected with the scrambled negative control which were treated with 20nM of paclitaxel exhibited a decrease in cell viability of 55.4% and 56.3% respectively. While untransfected cells or cells transfected with the scrambled negative control siRNA which were treated with 1μM of paclitaxel exhibited a decrease in cell viability of 66.2% and 66.4% respectively. Additionally, despite there being no visual signs of cytotoxicity, untreated cells transfected with MAD2 siRNA also exhibited a 19% significant reduction in cell viability compared to untransfected cells which were untreated potentially indicating a reduction in cell proliferation. Furthermore, in transfected cells treated with either dose of paclitaxel minimal if any visual signs of cytotoxicity were observed. In fact, the addition of paclitaxel appeared to accelerate the timeframe for the emergence of the enlarged cell phenotype. Additionally, the difference in cell viability between cells transfected with MAD2 siRNA which were untreated and transfected cells treated with either dose of paclitaxel was not statistically significant. This result was further supported by a trypan blue exclusion assay which detected a significant decrease in the number of dead cells in the supernatants of paclitaxel treated cells following knockdown of MAD2 compared to controls (Fig 4E). The results indicated that SKOV-3 cells transfected with siRNA targeting MAD2 were rendered resistant to paclitaxel and potentially undergoing cellular senescence. In order to investigate this further, SKOV-3 cells were stained with the senescence β-galactosidase staining kit. The number of cells which were β-galactosidase positive were counted and then compared against background levels in negative control and untreated cells (Fig 4B). A three-fold increase in the percentage of β-galactosidase positive cells was observed following knockdown of MAD2 compared to the untransfected and scramble negative controls (p<0.001), which was sufficient to indicate the induction of cellular senescence (Fig 4D). We also assessed the cytotoxicity of SKOV-3 cells to carboplatin following knockdown of either TLR4 or MAD2. Cells were transfected for 24 hours and then treated with a 200μM dose of carboplatin or left untreated, however, neither knockdown of TLR4 or MAD2 altered the response of SKOV-3 cells to carboplatin (Fig 4F).

**Fig 5:**
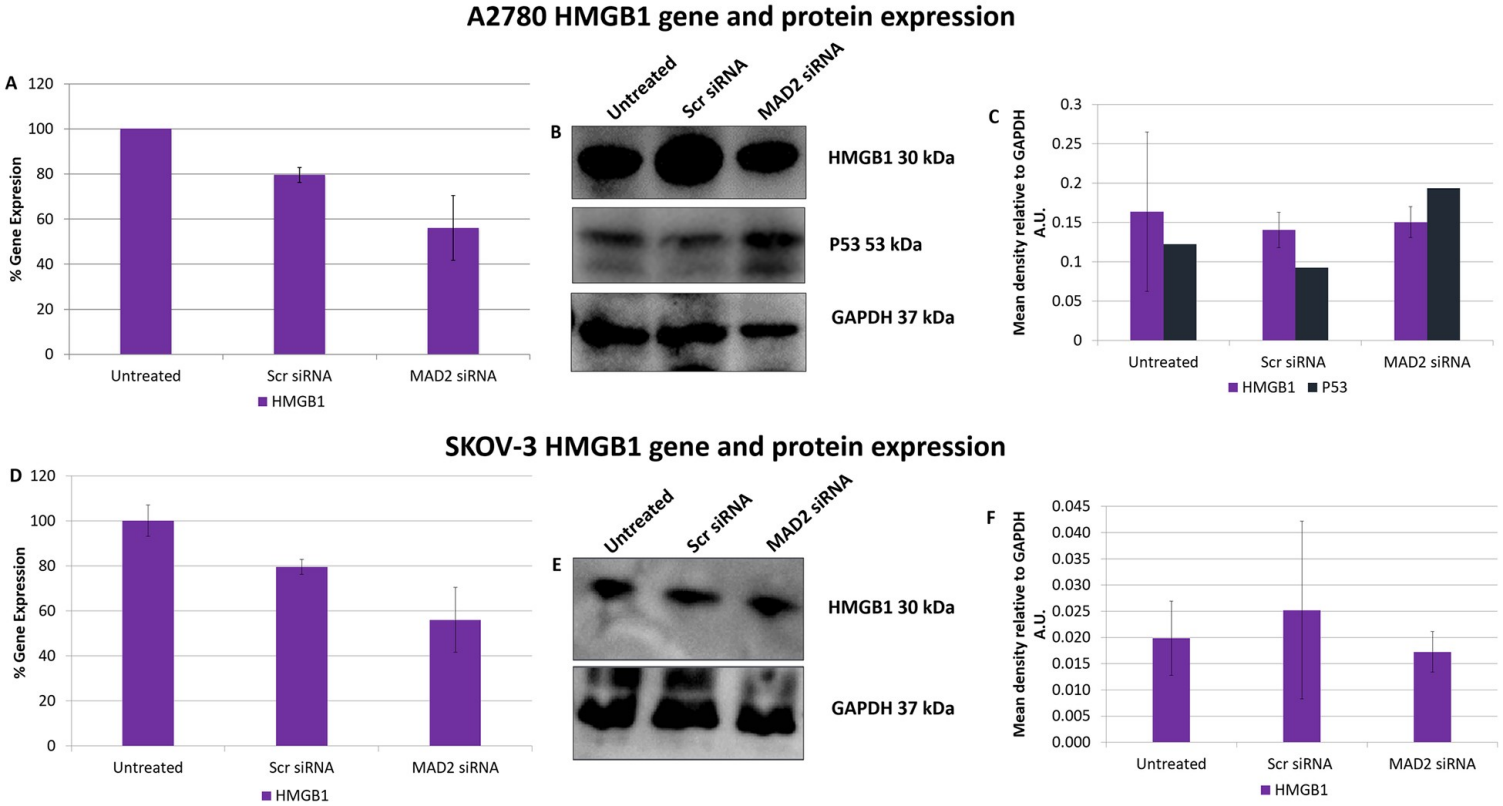
SiRNA knockdown of MAD2 does not alter HMGB1 gene or protein expression in A2780 or SKOV-3 cells. HMGB1 gene expression levels in A2780 (A) and SKOV-3 cells (D) following a 72 hour knockdown of MAD2. HMGB1 and P53 protein expression was also examined in A2780 cells using western blot analysis (B) and densitometry (C) while HMGB1 protein expression only was examined in P53 null SKOV-3 cells using western blot analysis (E) and densitometry (F). No significant change in HMGB1 gene or protein expression was observed in SKOV-3 cells nor was any change observed in HMGB1 gene expression or HMGB1 or P53 protein expression in A2780 cells post knockdown of MAD2 for 72 hours. Results are expressed as mean +/-SD. **Note**:- Blots are cropped from original images available in the S1 Raw Images.

Following these interesting results, High Mobility Group Box 1 (HMGB1) gene and protein expression was assessed post knockdown of MAD2 (Fig 5). HMGB1 was investigated as it had previously been shown to directly upregulate TLR4 expression [29] and due to the fact that its secretion during the early stages of senescence is known to be key to the formation of a SASP [30, 31]. However, no difference in HMGB1 expression was detected in either cell line and further evidence needs to be gathered to support the hypothesis that HMGB1 acts as the link between MAD2 mediated senescence and TLR4 upregulation.

### Microarray analysis

**Fig 6:**
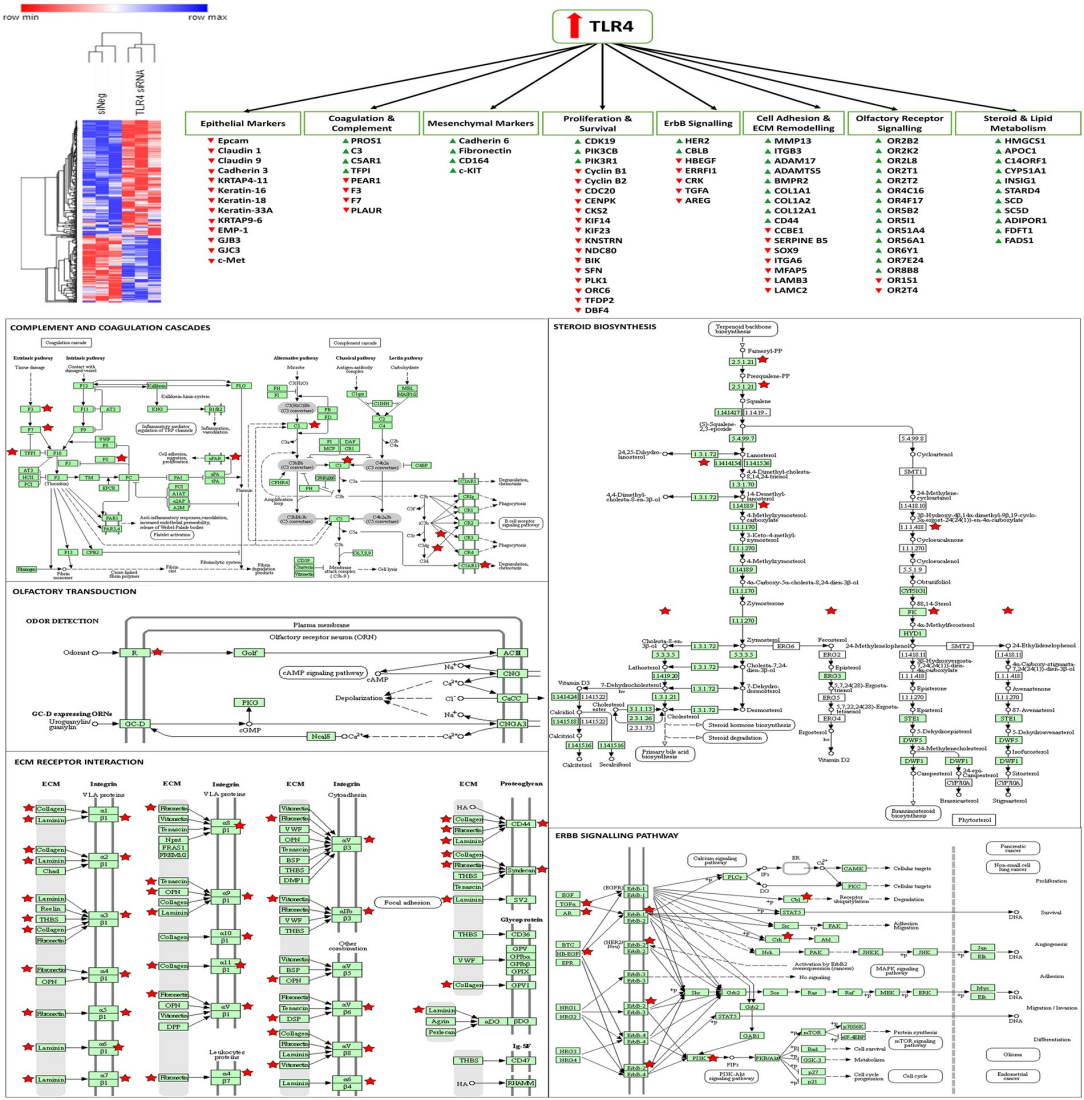
Altered genes and biological processes following knockdown of TLR4. Microarray analysis revealed that TLR4 controls genes related to EMT, survival and proliferation, steroid and lipid metabolism, olfactory receptor signalling, adhesion, coagulation and complement cascades, and ErbB signalling. A hierarchial clustering heatmap was generated using Morpheus from the broad institute. KEGG pathway maps were generated using DAVID, red stars indicate genes significantly altered in the TLR4 knockdown microarray data set.

As the knockdown of TLR4 had been shown to enhance the sensitivity of SKOV-3 cells to paclitaxel and the knockdown of MAD2 had been shown to render these cells paclitaxel resistant it was decided to perform microarray analysis post knockdown of TLR4 or MAD2 in this cell model. This was done in order to further discern any links between these biomarkers and gain greater insight into how they modulate the cellular response to paclitaxel. Following knockdown of TLR4 a total of 166 protein coding targets were found to be significantly upregulated and 286 targets found to be significantly (S1 Data). The differentially expressed genes identified following knockdown of TLR4 were subsequently analysed using the online gene ontology database DAVID, in order to identify important biological processes in which these genes participate [32]. A number of important biological processes were highlighted including cell death, cell adhesion, steroid biosynthesis and metabolism, complement and coagulation cascades and ErbB signalling among others (Fig 6 and S1 Table).

**Fig 7:**
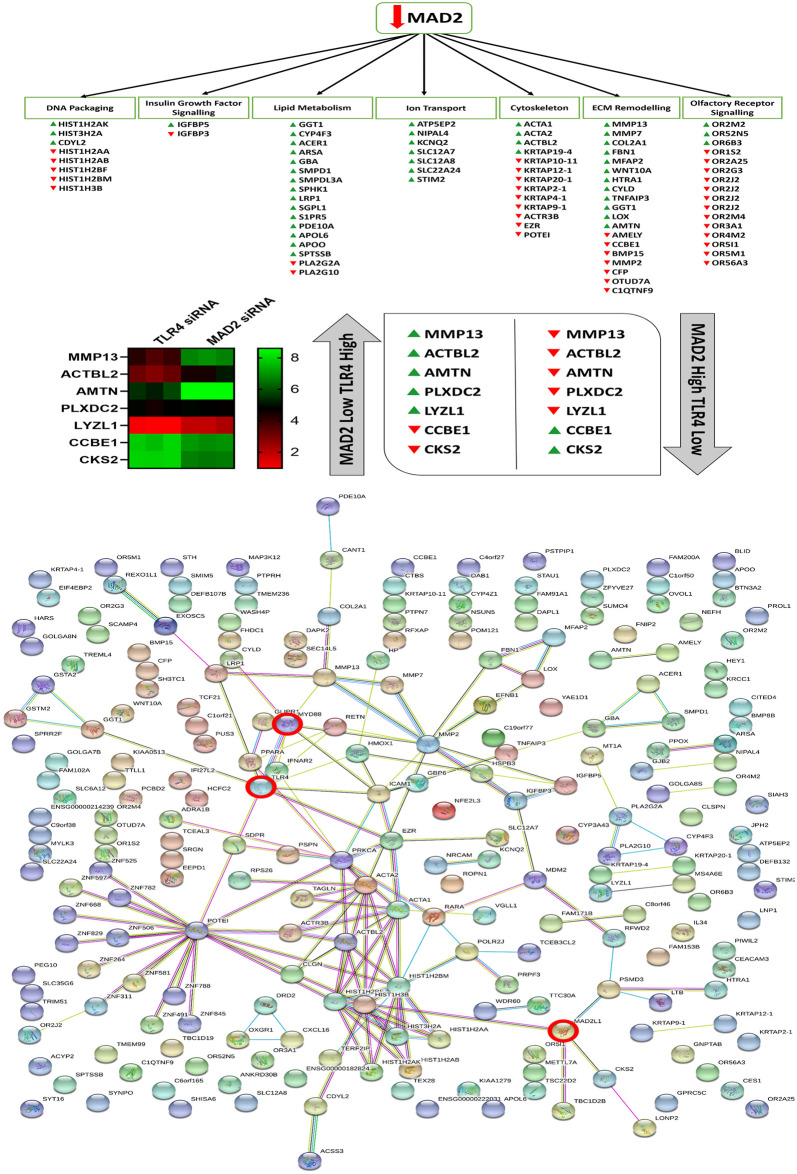
Altered genes and biological processes following knockdown of MAD2, string network analysis and cross comparison of the MAD2 and TLR4 knockdown data sets. Microarray analysis revealed that suppression of MAD2 and the induction of senescence affects various genes involved in DNA packaging, lipid metabolism, ion transport, Insulin-like growth factor signalling, ECM remodelling and olfactory receptor signalling and components of the actin cytoskeleton. Cross comparison of the two microarray data sets identified 7 key genes which are differentially regulated in both data sets. String network analysis plots of each of the altered genes identified in both data sets and how they relate to each other and TLR4, MyD88 and MAD2 which are highlighted by red circles.

Following knockdown of MAD2,126 protein coding genes were found to be upregulated and 95 protein coding genes were found to be downregulated (S2 Data). Microarray analysis highlighted several features of senescence, which were deregulated following knockdown of MAD2. These included an effect on DNA packaging, lipase activity, ion transporter activity, Insulin-like growth factor binding protein (IGFBP) activity, arachidonic acid metabolism, regulation of cell motility and migration, ossification and bone metabolism, the sensory perception of smell and the response to hormones and various chemical and extracellular stimuli (S2 Table). The complete microarray data sets for both knockdown experiments are available at ArrayExpress (Accession #370077). Cross-comparison of the microarray data sets highlighted 12 common genes which were deregulated in both data sets. This can be observed along with a map of the entire network of differentially expressed genes highlighted in both data sets in (Fig 7).

## Discussion

Our group and others have previously demonstrated that TLR4, MyD88 and MAD2 play key roles in paclitaxel resistance in ovarian cancer and are associated with poor patient outcome [3, 4, 6–9, 11–13, 15, 16, 24, 25, 33–36]. Although the recent and impressively sized study by Block et al. [24] demonstrated that TLR4 was not prognostic, several other studies including our own have previously demonstrated that TLR4 is linked to poor patient outcome and this trend can even be observed in the wider cancer space, with similar patterns observed in breast [10, 37], oesophageal [38] and other cancer types [39]. Block and colleagues [24] did find however that the TLR4 downstream adaptor protein MyD88 was prognostic, which is the signalling arm of the TLR4 pathway used to modulate the response of SKOV-3 cells to paclitaxel. This study sought to further explore how the TLR4-MyD88 signalling pathway and MAD2 mediated senescence contribute to the cellular response to paclitaxel and discern any molecular link between these three biomarkers as well as identifying new markers for potential future therapeutic exploitation. TLR4 itself is potentially targetable, a previous *in-vitro* study found that targeting TLR4-MyD88 signalling using the small molecule Atractylenolide-I could resensitise cells to paclitaxel [16]. Other TLR4 antagonists such as TAK-242 and Eritoran have also been examined in clinical trials for sepsis/inflammation as reviewed in [40] and may be suitable for ovarian cancer patients overexpressing TLR4 or MyD88. MyD88 is also targetable and such treatments may potentially eradicate paclitaxel resistant cancer stem cells (CSCs) by inducing differentiation [41, 42]. A wide array of inhibitors are also available for the TLR4-MyD88 pathway downstream transcription factor NFκB [43]. In this study suppression of TLR4 or alteration of MyD88 expression in either SKOV-3 or A2780 cells had no resulting impact on MAD2 expression. Intererestingly however in the OVCAR-3 serous ovarian model a significant increase in MAD2 expression was observed. One observation with this experiment was that the replicate with the highest knockdown of TLR4 had the highest upregulation of MAD2. Furthermore with both the SKOV-3 and A2780 cells lines MAD2 expression was partially increased ~1.5 fold post knockdown of TLR4, however this was not statistically significant. The observed differences may indicate that TLR4 needs to be supressed below a certain threshold to have a substantial impact on MAD2 expression. Increasing the efficacy of the TLR4 knockdown, through the use of shRNA vectors and cell selection or the use of newer technologies such as CRISPR may yield more definitive evidence for this.

Furthermore, suppression of MAD2 expression using siRNA in A2780, OVCAR-3 and SKOV-3 cell lines led to a significant increase in TLR4 gene expression levels demonstrating a key link between TLR4 and MAD2. Although it must be acknowledged that although TLR4 gene expression was upregulated post knockdown of MAD2, there was no recipient increase at the protein level in SKOV-3 or A2780 cells. This is a curious result, although TLR4 protein expression following knockdown of MAD2 was only examined at a single timepoint, 72 hours post transfection in both cell lines. However, there are two potential biological mechanisms which could explain why the recipient increase in TLR4 expression at the protein level was not observed. Firstly, the activation of the unfolded protein response as is known to occur during cellular senescence may have blocked or limited the amount of protein translation taking place [44]. Secondly, TLR4 shedding can occur as a result of oxidative stress [45] and during cellular senescence reactive oxygen species (ROS) levels are known to be dramatically increased [27]. Indeed, TLR4 shedding may even represent a feedback mechanism to blunt hyper-reactive TLR4-ligand signalling which may occur due to HMGB1 release which also occurs during senescence [31, 46]. HMGB1 was examined as part of this study however its expression was not found to be altered post knockdown, which may be as a result of its function as a secreted cytokine. TLR4 is also known to be upregulated by various other cytokines which may be secreted as part of the SASP [47–49].

A number of interesting target genes were highlighted during the microarray analysis in SKOV-3 cells following knockdown of TLR4 associated with metastasis, angiogenesis, EMT/differentiation and circulating tumour cell (CTC) biology including CD44, HER2, PI3K, MMP13, members of the claudin, cadherin, integrin and laminin family and various olfactory receptors (ORs). Although these markers require further validation, many of them have previously been shown to be prognostic for ovarian and other types of cancer and are potentially targetable [41, 42, 50–54]. TLR4 also appears to be suppressing both coagulation and complement which again may contribute to metastasis and therapeutic resistance [55–62]. Targeting of another marker identified on the arrays in the steroid biosynthesis group, farnesyl transferase has also previously been shown to enhance paclitaxel sensitivity in ovarian models [63] and has previously been targeted in clinical trials [64]. Some of the other steroid pathway targets have been shown to control vesicular trafficking of HER2 and related family members [65]. The microarray analysis indicates that the TLR4-MyD88 signalling pathway is likely driving paclitaxel resistance by promoting the induction of an EMT/stem like phenotype. Ligation of TLR4 by paclitaxel also likely provides a further survival advantage to these cells by upregulating pro-survival signalling molecules including pAKT, BCL-2, BCL-XL and XIAP as reported previously [7, 8, 37]. Given the crosstalk identified between MAD2 and TLR4 in this study it is likely that senescence is also helping to drive/amplify this phenomenon. Clearly new therapeutic strategies which actively target these mechanisms need to be introduced in order to enhance the efficacy of current ovarian cancer therapy.

Knockdown of MAD2 in the SKOV-3 cell model revealed a number of altered senescence associated genes and processes [27, 66–72]. Among those affected were genes involved in OR activity and the response to a number of different chemical and extracellular stimuli, IGFBP activity and ossification, cell motility, lipase & phospholipase activity and arachidonic acid metabolism, DNA packaging and ion transporter activity. Senescent cells likely act as a protective barrier against paclitaxel and perhaps other chemotherapeutic agents, shielding non-senescent populations of cancer cells such as CSCs from drug-induced cell death while simultaneously promoting their growth through the milieu of cytokines they release as part of the SASP [26, 27]. Previous reports have demonstrated that senescent cells are capable of promoting the growth of tumours and induce the progression of pre-malignant lesions into malignant tumours in *in-vivo* xenografts [73, 74]. They may also promote tumour growth through active suppression of immune cell populations [75]. Thus, senescence and an activated TLR4 signalling pathway likely promotes tumour growth through the generation of inflammatory niche which selects for invasive paclitaxel resistant CSC populations leading to shorter survival time in patients. Cross-comparison of microarray data sets highlighted several genes involved in cell adhesion, proliferation, differentiation, migration and extracellular matrix (ECM) degradation [76–86]. Targeting some of the markers highlighted in the arrays or treating patients with exogenous MAD2 may help to reverse the senescence phenotype and restore paclitaxel sensitivity. We also previously identified that the MAD2 regulatory microRNA miR-433 was dysregulated and associated with poor prognosis in ovarian cancer patients and may act as an upstream inducer of MAD2 mediated senescence [11]. Therefore, these patients may benefit from miR-433 antagomir therapy. A variety of other anti-senescence therapies are available which may help boost paclitaxel efficacy when MAD2 or miR-433 are used as triage markers [26, 87]. The results also demonstrate that these senescent cell populations appear to be selectively resistant to paclitaxel but are sensitive to carboplatin, therefore patients could potentially be selected out for single arm therapy with carboplatin.

## Conclusions

The molecular link between TLR4-MyD88 signalling and MAD2 identified in this study has potentially important implications for the development of new treatment strategies for ovarian cancer patients. Individually these markers highlight paclitaxel resistance mechanisms within a patient’s tumour. Depending on the expression of these markers, one or multiple mechanisms may need to be targeted. The complexity of downstream signalling pathways identified by microarray analysis also further highlight the fact that a single biomarker alone may be insufficient to capture the multiple pathophysiological processes occurring within a patient’s tumour which contribute to chemoresistance. Assessing multiple biomarkers such as TLR4, MyD88 and MAD2 which give greater insight into the pathological makeup of a patient’s tumour may help to direct therapies and more suitable treatment combinations in order to improve overall outcome.

## Methods

### Cell culture

A2780, OAW42, OVCAR-3, PEO1 and KURAMOCHI cells were cultured in RPMI 1640 medium (Sigma Aldrich, St Louis, USA), 59M cells were cultured in DMEM (Sigma Aldrich) and SKOV-3 cells were cultured in McCoys modified 5A medium (Sigma Aldrich) respectively. All media was supplemented with 10% foetal bovine serum (FBS) (Sigma Aldrich) and 2% penicillin/streptomycin (5000IU, Sigma Aldrich) and cells were maintained in a humidified atmosphere at 37°C and 5% CO_2_.

### Small interfering RNA transfection

siRNA targeting TLR4 (TLR4 siRNA, s14194), MyD88 (MyD88 siRNA, s9136) and silencer select negative control #1 siRNA (siNeg, 4390843) were purchased from (Thermo Fisher Scientific, Waltham, USA) and on target plus SMARTpool MAD2L1 siRNA (MAD2 siRNA, L-003271-00-0005) and on target plus SMARTpool non-targeting siRNA (Scr siRNA, D-001810-01-05) were purchased from (Dharmacon, Lafayette, USA). SKOV-3 cells were transfected into either 24 or 6 well plates at seeding densities of 25,000 or 125,000 cells per well respectively. Cells were transfected with Lipofectamine RNAiMAX (13778–075, Thermo Fisher Scientific), Opti-MEM^®^ I reduced serum medium (31985–047, Thermo Fisher Scientific) and siRNA at a final concentration of 1nM per well. A2780 cells were transfected into 6 well plates at a seeding density of 400,000 cells per well and were transfected with siRNA targeting TLR4 or MAD2 at final concentrations of 10nM or 30nM per well respectively. OVCAR-3 cells were transfected into 24 well plates at a seeding density of 25,000 cells per well. OVCAR-3 cells were transfected with 30nM of siRNA targeting TLR4 or MAD2. All transfections were carried out using media not containing antibiotics.

### MyD88 transfections

For the MyD88 transfection experiments A2780 cells were transfected with a MyD88 overexpression plasmid (MyD88 OE) or an empty vector negative control plasmid (eV Control), both purchased from IMAgenes, or were left untreated for 72 hours. For each transfection experiment, plasmid DNA and lipofectamine was first diluted in Opti-MEM^®^ I reduced serum medium. A2780 cells were transfected into 6 well plates at a seeding density of 400,000 cells per well. All transfections were carried out using media not containing antibiotics. The final plasmid DNA concentration per well was 1ng/ul.

### RNA extraction and TaqMan RT-PCR

Total RNA was isolated as per the manufacturer’s instructions using the *mir*Vana^™^ miRNA Isolation Kit (AM1560, Thermo Fisher Scientific). RNA concentration was determined using a nanodrop 2000c spectrophotometer (Thermo Fisher Scientific). Reverse transcription was carried out using the High Capacity cDNA Reverse Transcription Kit (4368813, Thermo Fisher Scientific) on the Gene Amp PCR System 9600 (Perkin Elmer, Waltham, USA). TaqMan RT-PCR was then performed using the 7900HT Real-Time PCR System (Thermo Fisher Scientific). Primers and probes for TLR4 (Hs00152939_m1), MAD2 (H203063324_g1), MyD88 (Hs00182082_m1), HMGB1 (Hs01037385_s1) and the endogenous controls, glyceraldehyde 3-phosphate dehydrogenase (GAPDH, 4333764T) or Beta-2 Microglobulin (B2M, 4333766F) were obtained from (Thermo Fisher Scientific). These are supplied as commercial pre-designed primer and probe mixes (20X). Gene expression levels following transfection were calculated using the ΔΔCT method relative to the endogenous control [88]. A significant change in gene expression was considered to be present if at least a 2-fold change (above 200% expression or below 50% expression) in gene expression was observed, with a p value of ≤0.05 compared to untreated cells and/or negative control cells.

### Western blot analysis

Following transfection for 72 hours protein was extracted from SKOV-3 cells using RIPA lysis buffer (Sc-24948, Santa Cruz Biotechnology, Santa Cruz, USA) modified with phenylmethanesulfonyl fluoride (PMSF) (200mM), a protease inhibitor cocktail, and sodium orthovanadate (Na_3_VO_4_) (100mM). Cell suspensions were later sonicated to ensure complete lysis using the soniprep 150 (MSE Labs, East Sussex, UK). Protein concentration was then determined using the Pierce^™^ BCA Protein Assay Kit (23225, Thermo Fisher Scientific). 30μg of protein samples were then resolved by SDS-PAGE on 4–12% Bis-Tris NuPage gels (NP0321, Thermo Fisher Scientific) using the XCell SureLock^®^ Mini-Cell SDS PAGE rig (Thermo Fisher Scientific). Resolved proteins were then transferred to 0.2μM Hybond PVDF membranes (10600021, Amersham, Amersham, UK) using the XCell II^™^ Blot Module (Thermo Fisher Scientific). Following transfer membranes were blocked using 5% w/v milk protein and probed using antibodies directed against TLR4 (1:100, Ab47093, Abcam), MAD2 (1: 1000, 610679, BD Biosciences), MyD88 (1:1000, D80F5, Cell Signalling Technology), P53 (1:500, sc-126, Santa Cruz Biotechnology), HMGB1 (1:50, sc-56698, Santa Cruz Biotechnology) or GAPDH (1: 10,000, Ab9485, Abcam). After washing, the membrane was incubated with either a horseradish peroxidase (HRP) linked anti-rabbit secondary antibody (#7074, 1:1000, Cell Signalling Technology) or an anti-mouse HRP-linked secondary antibody (#7076, 1:1000, Cell Signalling Technology). Following incubation with the primary and secondary antibodies, a detection reagent luminol (SC-2048, Santa Cruz Biotechnology) was applied to blots and chemiluminescence images were then developed using a LAS-4000 luminescent image analyser (Fujifilm, Minato, Japan). Molecular weight was confirmed using a MagicMark^™^ XP Western Protein Standard (LC5602, Thermo Fisher Scientific) and SeeBlue^™^ Plus2 Pre-stained Protein Standard (LC5925, Thermo Fisher Scientific). Restore^™^ PLUS Western Blot Stripping Buffer (46430, Thermo Fisher Scientific) was used to remove bound primary and secondary antibodies from membranes so they could be reprobed with additional antibodies. Densitometry was then carried out using Quantity One software (Bio-Rad Laboratories, Hercules, USA). Abundance of protein in arbitrary units (A.U.) was normalised to GAPDH. The mean density ratio of triplicate bands for each condition was then determined.

### Senescence β-galactosidase staining kit

The induction of senescence in cells is usually accompanied by an increase in β-galactosidase activity [89]. In order to demonstrate this, cells were stained with the senescence β-galactosidase staining kit (#9860, Cell Signalling Technology) following transfection for 72 hours and 120 hours. Images were then taken at 10X magnification using an Olympus CKX41 microscope and an Olympus E600 camera (Olympus, Shinjuku, Japan). The percentage of β-galactosidase positive cells within each image was then calculated for each condition for (n = 3) technical and (n = 3) biological replicates.

### Drug treatment and assessment of cell viability

Carboplatin (C2538), Paclitaxel (T402) and DMSO (D2650) were purchased from Sigma Aldrich. Carboplatin was diluted in sterile nuclease free water to a concentration of 10mg/ml and stored at room temperature based on recommendations by the manufacturer. Paclitaxel was diluted in DMSO to a concentration of 50g/l (58.6mM) based on recommendations by the manufacturer aliquoted and stored at -20°C while DMSO was kept at room temperature. Aliquots of Carboplatin, Paclitaxel and DMSO were freshly diluted with media for each experiment to the desired working concentrations. Following transfection for 24 hours, SKOV-3 cells were either left untreated, treated with 0.0017% DMSO (vehicle control) or 20nM or 1μM of paclitaxel or 200μM of Carboplatin for 48 hours. Forty-eight hours post-treatment, cell viability was assessed using the cell cycle kit 8 (CCK-8) assay. Absorbance values were read at 450nm using the Sunrise^™^ microplate reader (Tecan Trading AG, Männedorf, Switzerland). Cell viability for each condition/drug treatment was calculated as a % of non-transfected cells which were left untreated. Images were taken at 4X magnification using an axiovert 35 inverted microscope (Zeiss, Germany) and at 6X zoom using a Canon Powershot A620 digital camera.

### Trypan blue dye exclusion assay

SKOV-3 cells were transfected into 6 well plates with siRNA targeting MAD2 a nontargeting scrambled negative control siRNA or were left untreated for 72 hours. After 72 hours, cells were left untreated, treated with DMSO or were treated with 20nM paclitaxel and incubated for a further 48 hours. After the 48-hour drug incubation time, supernatants from each well were collected and wells rinsed with PBS to remove any residual dying cells. Collected supernatant and washings were pelleted by centrifugation and resuspended in a small volume of PBS. Cell suspensions were then mixed at 1:1 ratio with trypan blue (T8154, Sigma Aldrich) and the number of dead cells were counted using a haemocytometer.

### Microarray analysis

Prior to analysing RNA samples using Affymetrix microarrays, the quality of RNA samples was assessed using the Agilent 2100 Bioanalyzer. Samples were run on chips from the RNA 6000 Nano kit (5067–1511, Agilent Technologies, Santa Clara, USA) and an RNA Integrity Number (RIN) was obtained. 250ng of each RNA sample was then converted into sense strand cDNA using the GeneChip^®^ WT PLUS Reagent Kit (902280, Affymetrix). Each cDNA sample was then hybridised to Affymetrix GeneChip^®^ human gene 2.0 ST arrays (902113, Affymetrix). Arrays were washed using the Affymetrix GeneChip^®^ fluidics station 450 and scanned using the Affymetrix GeneChip^®^ Scanner 3000. Gene array data was analysed using Bioconductor software libraries available at (www.bioconductor.org) [90] and the RMA method [91]. Differential expression analysis across all the arrays was carried out using RankProd [92]. DAVID, a free bioinformatics resource was used to characterise differentially expressed genes in order to identify molecular function and biological process-related genes through gene ontology [32]. Microarray analysis was performed using three biological replicates of SKOV-3 cells transfected with either the scrambled or negative control siRNA or siRNA targeting TLR4 or MAD2. A 1.5 fold change in gene expression and a p value of <0.05 was set as the threshold for a significantly upregulated/downregulated gene, this threshold is in line with other published works [93]. Heatmaps were generated using Morpheus (https://software.broadinstitute.org/morpheus) or Graphpad Prism v8.4. KEGG pathway maps were generated using DAVID. The Affymetrix microarray data sets generated as part of this study are available in an ArrayExpress repository, accession #370077.

### *In-silico* analysis

*In-silico* analysis was performed in order to identify any potential interaction between the TLR4-MyD88 pathway and MAD2 using the Search Tool for the Retrieval of Interacting Genes/Proteins (STRING) v10 software which is freely available at (http://string-db.org/). This free online bioinformatics resource identifies protein-protein interactions through both direct (physical) as well as indirect (functional) associations [28].

### Statistical analysis

A student’s t-test was performed on all qPCR, densitometry and cell viability data to assess the statistical significance of gene silencing experiments and differences in cell viability between drug-treated versus untreated and vehicle control groups. A statistically significant difference was considered to be present at p≤0.05. Statistical analysis was performed using Microsoft Excel 2016.

## Supporting information

S1 Data List of differentially expressed genes following siRNA knockdown of TLR4 in SKOV-3 cells. (XLSX)

S2 Data List of differentially expressed genes following siRNA knockdown of MAD2 in SKOV-3 cells. (XLSX)

S1 Raw Images Uncropped western blots from A2780 and SKOV-3 cells. (PDF)

S1 File IC50 data for A2780 and SKOV-3 cells. (DOCX)

S1 Table Significantly over-represented biological processes identified by the DAVID database following knockdown of TLR4 in SKOV-3 cells. (DOCX)

S2 Table Features of senescence highlighted by microarray analysis following knockdown of MAD2 in SKOV-3 cells for 72 hours. (DOCX)

## References

[1] Siegel RL, Miller KD, Jemal A. Cancer statistics, 2018. CA Cancer J Clin. 2018;68(1):7–30. 10.3322/caac.21442 29313949

[2] Kim A, Ueda Y, Naka T, Enomoto T. Therapeutic strategies in epithelial ovarian cancer. J Exp Clin Cancer Res. 2012 1;31(1):14 10.1186/1756-9966-31-14 22330607 PMC3309949

[3] Wang AC, Su QB, Wu FX, Zhang XL, Liu PS. Role of TLR4 for paclitaxel chemotherapy in human epithelial ovarian cancer cells. Eur J Clin Invest. 2009 3;39(2):157–64. 10.1111/j.1365-2362.2008.02070.x 19200169

[4] Sun NK, Huang SL, Chang TC, Chao CCK. TLR4 and NFκB signaling is critical for taxol resistance in ovarian carcinoma cells. J Cell Physiol. 2018;233(3):2489–501. 10.1002/jcp.26125 28771725

[5] Prencipe M, Fitzpatrick P, Gorman S, Tosetto M, Klinger R, Furlong F, et al Cellular senescence induced by aberrant MAD2 levels impacts on paclitaxel responsiveness in vitro. Br J Cancer. 2009 12;102(11):1900–8. 10.1038/sj.bjc.6605419 19935801 PMC2788249

[6] D’Adhemar CJ, Spillane CD, Gallagher MF, Bates M, Costello KM, Barry-O’Crowley J, et al The MyD88+ phenotype is an adverse prognostic factor in epithelial ovarian cancer. PLoS One. 2014;9(6). 10.1371/journal.pone.0100816 24977712 PMC4076208

[7] Szajnik M, Szczepanski MJ, Czystowska M, Elishaev E, Mandapathil M, Nowak-Markwitz E, et al TLR4 signaling induced by lipopolysacharide or paclitaxel regulates tumor survival and chemoresistance in ovarian cancer. Oncogene. 2009;28(49):4353–63. 10.1038/onc.2009.289 19826413 PMC2794996

[8] Kelly MG, Alvero AB, Chen R, Silasi D-A, Abrahams VM, Chan S, et al TLR-4 signaling promotes tumor growth and paclitaxel chemoresistance in ovarian cancer. Cancer Res. 2006 4;66(7):3859–68. 10.1158/0008-5472.CAN-05-3948 16585214

[9] Wang AC, Ma YB, Wu FX, Ma ZF, Liu NF, Gao R, et al TLR4 induces tumor growth and inhibits paclitaxel activity in MyD88-positive human ovarian carcinoma in vitro. Oncol Lett. 2014;7(3):871–7. 10.3892/ol.2013.1759 24527095 PMC3919821

[10] Haricharan S, Brown P. TLR4 has a TP53-dependent dual role in regulating breast cancer cell growth. Proc Natl Acad Sci. 2015;2015:201420811. 10.1073/pnas.1420811112 PMC4485113 26063617

[11] Furlong F, Fitzpatrick P, O’Toole S, Phelan S, McGrogan B, Maguire A, et al Low MAD2 expression levels associate with reduced progression-free survival in patients with high-grade serous epithelial ovarian cancer. J Pathol. 2012 4;226(5):746–55. 10.1002/path.3035 22069160 PMC3593171

[12] Byrne T, Coleman HG, Cooper JA, Mccluggage WG, Mccann A, Furlong F. The association between MAD2 and prognosis in cancer: A systematic review and meta-analyses. Oncotarget. 2017;8(60):102223–34. 10.18632/oncotarget.18414 29254238 PMC5731948

[13] Mcgrogan B, Phelan S, Fitzpatrick P, Maguire A, Prencipe M, Brennan D, et al Spindle assembly checkpoint protein expression correlates with cellular proliferation and shorter time to recurrence in ovarian cancer. Hum Pathol. 2014;45(7):1509–19. 10.1016/j.humpath.2014.03.004 24792619

[14] Byrne T, Nelson L, Beirne JP, Sharpe D, Quinn Jennifer E. McCluggage, et al BRCA1 and MAD2 are coexpressed and are prognostic indicators in tubo-ovarian high-grade serous carcinoma. Int J Gynecol Cancer. 2018;28(3):472–8. 10.1097/IGC.0000000000001214 29465507

[15] Kim KH, Jo MS, Suh DS, Yoon MS, Shin DH, Lee JH, et al Expression and significance of the TLR4/MyD88 signaling pathway in ovarian epithelial cancers. World J Surg Oncol. 2012 1;10(1):193 10.1186/1477-7819-10-193 22985132 PMC3539930

[16] Huang JM, Zhang G-N, Shi Y, Zha X, Zhu Y, Wang M-M, et al Atractylenolide-I sensitizes human ovarian cancer cells to paclitaxel by blocking activation of TLR4/MyD88-dependent pathway. Sci Rep. 2014 1;4:3840 10.1038/srep03840 24452475 PMC3899591

[17] Sato Y, Goto Y, Narita N, Hoon DSB. Cancer Cells Expressing Toll-like Receptors and the Tumor Microenvironment. Cancer Microenviron. 2009 9;2 Suppl 1:205–14. 10.1007/s12307-009-0022-y 19685283 PMC2756339

[18] Ran S. The role of TLR4 in chemotherapy-driven metastasis. Cancer Res. 2015;(10):2405–11. 10.1158/0008-5472.CAN-14-3525 25998620 PMC4470764

[19] Chen R, Alvero a B, Silasi D, Steffensen KD, Mor G. Cancers take their Toll--the function and regulation of Toll-like receptors in cancer cells. Oncogene. 2008 1;27(2):225–33. 10.1038/sj.onc.1210907 18176604

[20] Byrd-Leifer CA, Block EF, Takeda K, Akira S, Ding A. The role of MyD88 and TLR4 in the LPS-mimetic activity of Taxol. Eur J Immunol. 2001 8;31(8):2448–57. 10.1002/1521-4141(200108)31:8<2448::aid-immu2448>3.0.co;2-n 11500829

[21] Chen R, Alvero AB, Silasi DA, Mor G. Inflammation, cancer and chemoresistance: taking advantage of the toll-like receptor signaling pathway. Am J Reprod Immunol. 2007 2;57(2):93–107. 10.1111/j.1600-0897.2006.00441.x 17217363

[22] Huang B, Zhao J, Unkeless JC, Feng ZH, Xiong H. TLR signaling by tumor and immune cells: a double-edged sword. Oncogene. 2008 1;27(2):218–24. 10.1038/sj.onc.1210904 18176603

[23] Li Z, Block MS, Vierkant RA, Fogarty ZC, Winham SJ, Visscher DW, et al The inflammatory microenvironment in epithelial ovarian cancer: a role for TLR4 and MyD88 and related proteins. Tumor Biol. 2016;37(10):13279–86. 10.1007/s13277-016-5163-2 27460076 PMC5097682

[24] Block MS, Vierkant RA, Rambau PF, Winham SJ, Wagner P, Traficante N, et al MyD88 and TLR4 expression in epithelial ovarian cancer. Mayo Clin Proc. 2018 4;93(3):307–20. 10.1016/j.mayocp.2017.10.023 29502561 PMC5870793

[25] Silasi DA, Alvero AB, Illuzzi J, Kelly M, Chen R, Fu H-H, et al MyD88 predicts chemoresistance to paclitaxel in epithelial ovarian cancer. Yale J Biol Med. 2006 12;79(3–4):153–63. 17940625 PMC1994803

[26] Bates M, Furlong F, Gallagher MF, Spillane CD, McCann A, O’Toole S, et al Too MAD or not MAD enough: The duplicitous role of the spindle assembly checkpoint protein MAD2 in cancer. Cancer Lett. 2020;469(October 2019):11–21. 10.1016/j.canlet.2019.10.005 31593803

[27] Davalos AR, Coppe JP, Campisi J, Desprez PY. Senescent cells as a source of inflammatory factors for tumor progression. Cancer Metastasis Rev. 2010;29(2):273–83. 10.1007/s10555-010-9220-9 20390322 PMC2865636

[28] Szklarczyk D, Franceschini A, Wyder S, Forslund K, Heller D, Huerta-Cepas J, et al STRING v10: protein-protein interaction networks, integrated over the tree of life. Nucleic Acids Res. 2015;43:D447–52. 10.1093/nar/gku1003 25352553 PMC4383874

[29] Chen Y, Huang XJ, Yu N, Xie Y, Zhang K, Wen F, et al HMGB1 contributes to the expression of P-Glycoprotein in mouse epileptic brain through toll-like receptor 4 and receptor for advanced glycation end products. PLoS One. 2015;10(10). 10.1371/journal.pone.0140918 26485677 PMC4613137

[30] Salminen A, Kauppinen A, Kaarniranta K. Emerging role of NF-κB signaling in the induction of senescence-associated secretory phenotype (SASP). Vol. 24, Cellular Signalling. 2012 p. 835–45. 10.1016/j.cellsig.2011.12.006 22182507

[31] Davalos AR, Kawahara M, Malhotra GK, Schaum N, Huang J, Ved U, et al p53-dependent release of alarmin HMGB1 is a central mediator of senescent phenotypes. J Cell Biol. 2013;201(4):613–29. 10.1083/jcb.201206006 23649808 PMC3653366

[32] DennisGJ, ShermanB, HosackD, YangJ, GaoW, LaneH, et al DAVID: Database for annotation, visualization, and integrated discovery. Genome. 2003;4(5).12734009

[33] Luo XZ, He QZ, Wang K. Expression of Toll-like receptor 4 in ovarian serous adenocarcinoma and correlation with clinical stage and pathological grade. Int J Clin Exp Med. 2015;8(8):14323–7. 26550417 PMC4613102

[34] Park PE, Jeong JY, Kim SZ, Park JY. MAD2 expression in ovarian carcinoma: Different expression patterns and levels among various types of ovarian carcinoma and its prognostic significance in high-grade serous carcinoma. Korean J Pathol. 2013;47(5):418–25. 10.4132/KoreanJPathol.2013.47.5.418 24255629 PMC3830988

[35] Nakano Y, Sumi T, Teramae M, Morishita M, Fukuda T, Terada H, et al Expression of the mitotic-arrest deficiency 2 is associated with chemotherapy resistance in ovarian serous adenocarcinoma. Oncol Rep. 2012;28(4):1200–4. 10.3892/or.2012.1907 22797604

[36] Hao X, Zhou Z, Ye S, Zhou T, Lu Y, Ma D, et al Effect of Mad2 on paclitaxel-induced cell death in ovarian cancer cells. J Huazhong Univ Sci Technolog Med Sci. 2010 10;30(5):620–5. 10.1007/s11596-010-0553-y 21063845

[37] Rajput S, Volk-Draper LD, Ran S. TLR4 is a novel determinant of the response to paclitaxel in breast cancer. Mol Cancer Ther. 2013 8;12(8):1676–87. 10.1158/1535-7163.MCT-12-1019 23720768 PMC3742631

[38] Huhta H, Helminen O, Lehenkari PP, Saarnio J, Karttunen TJ, Kauppila JH. Toll-like receptors 1, 2, 4 and 6 in esophageal epithelium, Barrett’s esophagus, dysplasia and adenocarcinoma. Oncotarget. 2016;7(17):23658–67. 10.18632/oncotarget.8151 27008696 PMC5029654

[39] Hao B, Chen Z, Bi B, Yu M, Yao S, Feng Y, et al Role of TLR4 as a prognostic factor for survival in various cancers: A meta-analysis. Oncotarget. 2018;9(16):13088–99. 10.18632/oncotarget.24178 29560134 PMC5849198

[40] Kuzmich NN, Sivak K V., Chubarev VN, Porozov YB, Savateeva-Lyubimova TN, Peri F. TLR4 signaling pathway modulators as potential therapeutics in inflammation and sepsis. Vaccines. 2017;5(4):1–25. 10.3390/vaccines5040034 28976923 PMC5748601

[41] Sulaiman G, Cooke A, Ffrench B, Gasch C, Abdullai OA, O’Connor K, et al MyD88 is an essential component of retinoic acid-induced differentiation in human pluripotent embryonal carcinoma cells. Cell Death Differ. 2017;24(11):1975–86. 10.1038/cdd.2017.124 28885616 PMC5635222

[42] Alvero AB, Chen R, Fu H, Montagna M, Schwartz PE, Rutherford T, et al Molecular phenotyping of human ovarian cancer stem cells unravel the mechanisms for repair and chemo-resistance. Cell Cycle. 2011;8(1):158–66. 10.4161/cc.8.1.7533 PMC3041590 19158483

[43] Gilmore TD, Herscovitch M. Inhibitors of NF-kappaB signaling: 785 and counting. Oncogene. 2006;25(51):6887–99. 10.1038/sj.onc.1209982 17072334

[44] Pluquet O, Pourtier A, Abbadie C. The unfolded protein response and cellular senescence. A review in the theme: cellular mechanisms of endoplasmic reticulum stress signaling in health and disease. Am J Physiol—Cell Physiol. 2015;308(6):C415–25. 10.1152/ajpcell.00334.2014 25540175

[45] Zhang B, Ramesh G, Uematsu S, Akira S, Reeves WB. TLR4 signaling mediates inflammation and tissue injury in nephrotoxicity. J Am Soc Nephrol. 2008;19:923–32. 10.1681/ASN.2007090982 18256356 PMC2386719

[46] Yang WS, Kim JJ, Lee MJ, Lee EK, Park SK. Ectodomain shedding of RAGE and TLR4 as a negative feedback regulation in high-mobility group box 1-activated aortic endothelial cells. Cell Physiol Biochem. 2018;51(4):1632–44. 10.1159/000495651 30497069

[47] Tamandl D, Bahrami M, Wessner B, Weigel G, Ploder M, Fürst W, et al Modulation of toll-like receptor 4 expression on human monocytes by tumor necrosis factor and interleukin-6: Tumor necrosis factor evokes lipopolysacharide hyporesponsiveness, whereas interleukin-6 enhances lipopolysacharide activity. Shock. 2003;20(3):224–9. 10.1097/00024382-200309000-00005 12923493

[48] Greenhill CJ, Rose-John S, Lissilaa R, Ferlin W, Ernst M, Hertzog PJ, et al IL-6 trans-signaling modulates TLR4-dependent inflammatory responses via STAT3. J Immunol. 2010;186(2):1199–208. 10.4049/jimmunol.1002971 21148800

[49] Dias-Melicio LAA, Fernandes RK, Rodrigues DR, Golim MA, Soares AMVC, Maria A, et al Interleukin-18 increases TLR4 and mannose receptor expression and modulates cytokine production in human monocytes. Mediators Inflamm. 2015;2015:236839 10.1155/2015/236839 25873755 PMC4383410

[50] Wang W, Gao Y, Hai J, Yang J, Duan S. HER2 decreases drug sensitivity of ovarian cancer cells via inducing stem cell-like property in an NFκB-dependent way. Biosci Rep. 2019;39(3):1–12. 10.1042/BSR20180829 PMC6422889 30314995

[51] Sheng Q, Liu X, Fleming E, Yuan K, Piao H, Chen J, et al An activated ErbB3/NRG1 autocrine loop supports in vivo proliferation in ovarian cancer cells. Cancer Cell. 2010;17(3):298–310. 10.1016/j.ccr.2009.12.047 20227043 PMC2897158

[52] Kwon MJ. Emerging roles of claudins in human cancer. Int J Mol Sci. 2013;14(9):18148–80. 10.3390/ijms140918148 24009024 PMC3794774

[53] Kong D, Banerjee S, Ahmad A, Li Y, Wang Z, Sethi S, et al Epithelial to mesenchymal transition is mechanistically linked with stem cell signatures in prostate cancer cells. PLoS One. 2010;5(8). 10.1371/journal.pone.0012445 20805998 PMC2929211

[54] Zeng L, Qian J, Zhu F. The prognostic values of matrix metalloproteinases in ovarian cancer. J Int Med Res. 2019 10.1177/0300060519825983 31099295 PMC7140190

[55] Davies GF, Berg A, Postnikoff SDL, Wilson HL, Arnason TG, Kusalik A, et al TFPI1 mediates resistance to doxorubicin in breast cancer cells by inducing a hypoxic-like response. PLoS One. 2014;9(1). 10.1371/journal.pone.0084611 24489651 PMC3904823

[56] Iversen N, Lindahl AK, Abildgaard U. Elevated TFPI in malignant disease: relation to cancer type and hypercoagulation. Br J Haematol. 1998;102:889–95. 10.1046/j.1365-2141.1998.00875.x 9734635

[57] Asakura H, Ontachi Y, Mizutani T, Kato M, Saito M, Morishita E, et al Elevated levels of free tissue factor pathway inhibitor antigen in cases of disseminated intravascular coagulation caused by various underlying diseases. Blood Coagul Fibrinolysis. 2001 1;12(1):1–8. 10.1097/00001721-200101000-00001 11229821

[58] Arnason T, Harkness T. Development, maintenance, and reversal of multiple drug resistance: At the crossroads of TFPI1, ABC transporters, and HIF1α. Cancers (Basel). 2015;7(4):2063–82. 26501324 10.3390/cancers7040877 PMC4695877

[59] Tinholt M, Stavik B, Louch W, Carlson CR, Sletten M. Syndecan-3 and TFPI colocalize on the surface of endothelial-, smooth muscle-, and cancer cells. PLoS One. 2015;10(1):e0117404. 25617766 10.1371/journal.pone.0117404 PMC4305309

[60] Yoneda M, Imamura R, Nitta H, Taniguchi K, Saito F, Kikuchi KEN, et al Enhancement of cancer invasion and growth via the C5a-C5a receptor system: Implications for cancer promotion by autoimmune diseases and association with cervical cancer invasion. Oncol Lett. 2019;17:913–20. 10.3892/ol.2018.9715 30655847 PMC6313068

[61] Bareke H, Akbuga J. Complement system’s role in cancer and its therapeutic potential in ovarian cancer. Scand J Immunol. 2018;88(January):e12672. 29734524 10.1111/sji.12672

[62] Maher SG, McDowell DT, Collins BC, Muldoon C, Gallagher WM, Reynolds J V. Serum proteomic profiling reveals that pretreatment complement protein levels are predictive of esophageal cancer patient response to neoadjuvant chemoradiation. Ann Surg. 2011;254(5):809–17. 10.1097/SLA.0b013e31823699f2 22005152

[63] Marcus AI, O’Brate AM, Buey RM, Zhou J, Shala T, Khuri FR, et al Farnesyltransferase inhibitors reverse taxane resistance. Cancer Res. 2006;66(17):8838–46. 10.1158/0008-5472.CAN-06-0699 16951201 PMC1861825

[64] Meier W, Rau J, Gropp-meier M, Baumann K, Huober J, Wollschlaeger K, et al Randomized phase II trial of carboplatin and paclitaxel with or without lonafarnib in first-line treatment of epithelial ovarian cancer stage IIB-IV. Gynecol Oncol. 2012;126(2):236–40. 10.1016/j.ygyno.2012.04.050 22564713

[65] Sukhanova A, Gorin A, Serebriiskii IG, Gabitova L, Zheng H, Restifo D, et al Targeting C4-demethylating genes in the cholesterol pathway sensitizes cancer cells to EGF receptor inhibitors via increased EGF receptor degradation. Cancer Discov. 2013;3(1):96–112. 10.1158/2159-8290.CD-12-0031 23125191 PMC3546138

[66] Maßberg D, Jovancevic N, Offermann A, Simon A, Baniahmad A, Perner S, et al The activation of OR51E1 causes growth suppression of human prostate cancer cells. Oncotarget. 2016;3(30). 10.18632/oncotarget.10197 27374083 PMC5217014

[67] Nakayasu C, Kanemura F, Hirano Y, Shimizu Y, Tonosaki K. Sensitivity of the olfactory sense declines with aging in senescence-accelerated mouse (SAM-P1). Physiol Behav. 2000 7;70(1–2):135–9. 10.1016/s0031-9384(00)00234-1 10978488

[68] Farfariello V, Iamshanova O, Germain E, Fliniaux I, Prevarskaya N. Calcium homeostasis in cancer: A focus on senescence. Biochim Biophys Acta. 2014;1853(9):1974–9. 10.1016/j.bbamcr.2015.03.005 25764980

[69] Kim KS, Kim M-S, Seu YB, Chung HY, Kim JH, Kim J-R. Regulation of replicative senescence by insulin-like growth factor-binding protein 3 in human umbilical vein endothelial cells. Aging Cell. 2007;6(4):535–45. 10.1111/j.1474-9726.2007.00315.x 17635417

[70] Sharpless NE, Sherr CJ. Forging a signature of in vivo senescence. Nat Rev Cancer. 2015;15(7):397–408. 10.1038/nrc3960 26105537

[71] Pérez-Mancera PA, Young ARJ, Narita M. Inside and out: the activities of senescence in cancer. Nat Rev Cancer. 2014;14(8):547–58. 10.1038/nrc3773 25030953

[72] Lorenzini A, Hrelia S, Bordoni A, Biagi P, Frisoni L, Marinucci T, et al Is increased arachidonic acid release a cause or a consequence of replicative senescence? Exp Gerontol. 2001;36(1):65–78. 10.1016/s0531-5565(00)00192-3 11162912

[73] Liu D, Hornsby PJ. Senescent human fibroblasts increase the early growth of xenograft tumors via matrix metalloproteinase secretion. Cancer Res. 2007;67(7):3117–26. 10.1158/0008-5472.CAN-06-3452 17409418

[74] Coppé JP, Desprez PY, Krtolica A, Campisi J. The senescence-associated secretory phenotype: the dark side of tumor suppression. Annu Rev Pathol. 2010;5:99–118. 10.1146/annurev-pathol-121808-102144 20078217 PMC4166495

[75] Kaupp UB. Olfactory signalling in vertebrates and insects: differences and commonalities. Nat Rev Neurosci. 2010;11(3):188–200. 10.1038/nrn2789 20145624

[76] Krystosek A, Sachs L. Control of lysozyme induction in the differentiation of myeloid leukemic cells. Cell. 1976 12;9(4):675–84. 10.1016/0092-8674(76)90131-8 1017012

[77] Hödebeck M, Scherer C, Wagner A, Hecker M, Korff T. TonEBP/NFAT5 regulates ACTBL2 expression in biomechanically activated vascular smooth muscle cells. Front Physiol. 2014;5(Nov):1–8. 10.3389/fphys.2014.00467 25520667 PMC4253659

[78] Kitazono AA, Kron SJ. An essential function of yeast cyclin-dependent kinase Cdc28 maintains chromosome stability. J Biol Chem. 2002;277(50):48627–34. 10.1074/jbc.M207247200 12359726

[79] Guo JY, Hsu HS, Tyan SW, Li FY, Shew JY, Lee WH, et al Serglycin in tumor microenvironment promotes non-small cell lung cancer aggressiveness in a CD44-dependent manner. Oncogene. 2017;36(17):2457–71. 10.1038/onc.2016.404 27819672 PMC5415946

[80] Hishida T, Naito K, Osada S, Nishizuka M, Imagawa M. Peg10, an imprinted gene, plays a crucial role in adipocyte differentiation. FEBS Lett. 2007;581(22):4272–8. 10.1016/j.febslet.2007.07.074 17707377

[81] D’Amico L, Patanè S, Grange C, Bussolati B, Isella C, Fontani L, et al Primary breast cancer stem-like cells metastasise to bone, switch phenotype and acquire a bone tropism signature. Br J Cancer. 2013;108(12):2525–36. 10.1038/bjc.2013.271 23801032 PMC3694250

[82] Miake J, Notsu T, Higaki K, Hidaka K, Morisaki T, Yamamoto K, et al Cited4 is related to cardiogenic induction and maintenance of proliferation capacity of embryonic stem cell-derived cardiomyocytes during in vitro cardiogenesis. PLoS One. 2017;12(8):1–14. 10.1371/journal.pone.0183225 28817660 PMC5560578

[83] Pivetta E, Scapolan M, Pecolo M, Wassermann B, Abu-Rumeileh I, Balestreri L, et al MMP-13 stimulates osteoclast differentiation and activation in tumour breast bone metastases. Breast Cancer Res. 2011;13(5):R105 10.1186/bcr3047 22032644 PMC3262218

[84] Barton CA, Gloss BS, Qu W, Statham AL, Hacker NF, Sutherland RL, et al Collagen and calcium-binding EGF domains 1 is frequently inactivated in ovarian cancer by aberrant promoter hypermethylation and modulates cell migration and survival. Br J Cancer. 2010;102(1):87–96. 10.1038/sj.bjc.6605429 19935792 PMC2813742

[85] Lacruz RS, Nakayama Y, Holcroft J, Nguyen V, Somogyi-Ganss E, Snead ML, et al Targeted overexpression of amelotin disrupts the microstructure of dental enamel. PLoS One. 2012;7(4). 10.1371/journal.pone.0035200 22539960 PMC3335167

[86] Cheng G, Zhong M, Kawaguchi R, Kassai M, Al-Ubaidi M, Deng J, et al Identification of PLXDC1 and PLXDC2 as the transmembrane receptors for the multifunctional factor PEDF. Elife. 2014;3:e05401 10.7554/eLife.05401 25535841 PMC4303762

[87] Short S, Fielder E, Miwa S, von Zglinicki T. Senolytics and senostatics as adjuvant tumour therapy. EBioMedicine. 2019;41:683–92. 10.1016/j.ebiom.2019.01.056 30737084 PMC6441870

[88] Schmittgen TD, Livak KJ. Analyzing real-time PCR data by the comparative C(T) method. Nat Protoc. 2008;3(6):1101–8. 10.1038/nprot.2008.73 18546601

[89] Dimri GP, Leet X, Basile G, Acosta M, Scorrt G, Roskelley C, et al A biomarker that identifies senescent human cells in culture and in aging skin in vivo. 1995;92(September):9363–7. 10.1073/pnas.92.20.9363 7568133 PMC40985

[90] Gentleman RC, Carey VJ, Bates DM, Bolstad B, Dettling M, Dudoit S, et al Bioconductor: open software development for computational biology and bioinformatics. Genome Biol. 2004;5(10):R80 10.1186/gb-2004-5-10-r80 15461798 PMC545600

[91] Carvalho BS, Irizarry RA. A framework for oligonucleotide microarray preprocessing. Bioinformatics. 2010;26(19):2363–7. 10.1093/bioinformatics/btq431 20688976 PMC2944196

[92] Breitling R, Armengaud P, Amtmann A, Herzyk P. Rank products: A simple, yet powerful, new method to detect differentially regulated genes in replicated microarray experiments. FEBS Lett. 2004;573(1–3):83–92. 10.1016/j.febslet.2004.07.055 15327980

[93] Vathipadiekal V, Wang V, Wei W, Waldron L, Drapkin R, Gillette M, et al Creation of a human secretome: A novel composite library of human secreted proteins: Validation using ovarian cancer gene expression data and a virtual secretome array. Clin Cancer Res. 2015;(5):1–11. 10.1158/1078-0432.CCR-14-3173 25944803

