# Supplementary material for: The role of the MAD2-TLR4-MyD88 axis in paclitaxel resistance in ovarian cancer
Source: PLoS One. 2020 Dec 28;15(12):e0243715. doi: 10.1371/journal.pone.0243715 (PMC7769460; doi:10.1371/journal.pone.0243715)
Supplement: S1 Raw Images — (PDF) [file pone.0243715.s003.pdf]

The role of the MAD2-TLR4-MyD88 axis in paclitaxel resistance in ovarian cancer

Mark Bates PhD[1,2,3,4]*, Cathy D Spillane PhD[1,2,3], Michael F Gallagher PhD[1,2,3], Amanda McCann PhD[5], Cara Martin PhD[1,2,3,6], Gordon Blackshields PhD[2,3,6], Helen Keegan PhD[1,2,3,6], Luke Gubbins PhD[5], Robert Brooks PhD[7], Doug Brooks PhD[7], Stavros Selemidis[8], Sharon O'Toole PhD[1,2,3,4¶], John J O'Leary MD, PhD[1,2,3,6¶]

[1] Department of Histopathology, Trinity College Dublin, Dublin, Ireland

[2] Emer Casey Molecular Pathology Research Laboratory, Coombe Women & Infants University Hospital, Dublin, Ireland

[3] Trinity St James's Cancer Institute, Dublin, Ireland

[4] Department of Obstetrics and Gynaecology, Trinity College Dublin, Dublin, Ireland

[5] College of Health Sciences, University College Dublin, Belfield, Dublin, Ireland

[6] Department of Pathology, Coombe Women & Infants University Hospital, Dublin, Ireland

[7] School of Pharmacy and Medical Sciences, University of South Australia, Adelaide, Australia

[8] School of Health and Biomedical Sciences, Royal Melbourne Institute of Technology, Bundoora, Australia

¶Joint Senior Author
*Corresponding author

Email: batesm1@tcd.ie (MB)

Uncropped Western Blots

Western Blot Results Figure 1D

Protein lysates used in Figure 1D were initially probed for GAPDH, MyD88 and TLR4 to confirm knockdown of TLR4 and MyD88 in SKOV-3 cells in our previous PLOS One publication "The MyD88+ Phenotype Is an Adverse Prognostic Factor in Epithelial Ovarian Cancer" (PMID: 24977712) before subsequent reprobing with an antibody directed against MAD2 to determine whether disruption of TLR4/MyD88 signalling influenced protein expression levels of MAD2.

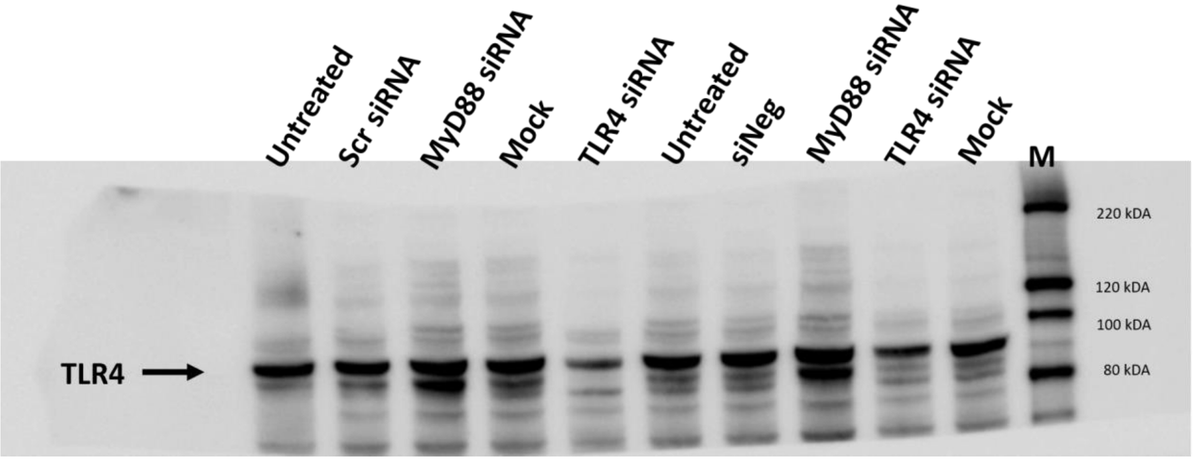

TLR4 protein expression in SKOV-3 cells following knockdown of MyD88 or TLR4. The blot was exposed for 140 seconds for optimum results. The band corresponding to TLR4 is indicated by the arrow. This blot represents the first two of four replicates.

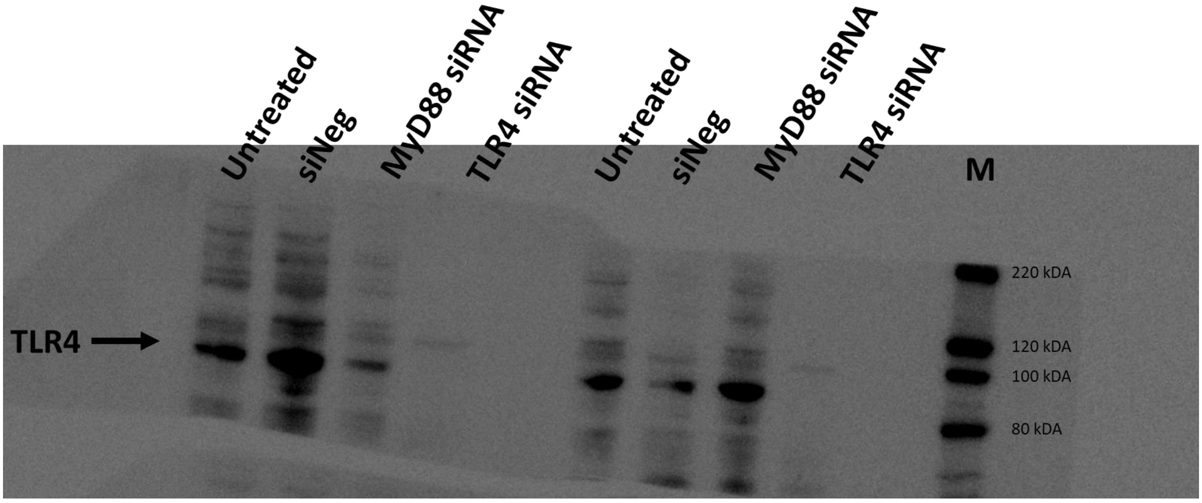

TLR4 protein expression in SKOV-3 cells following knockdown of MyD88 or TLR4. The blot was exposed for 140 seconds for optimum results. The band corresponding to TLR4 is indicated by the arrow. This blot represents the third and fourth of four replicates.

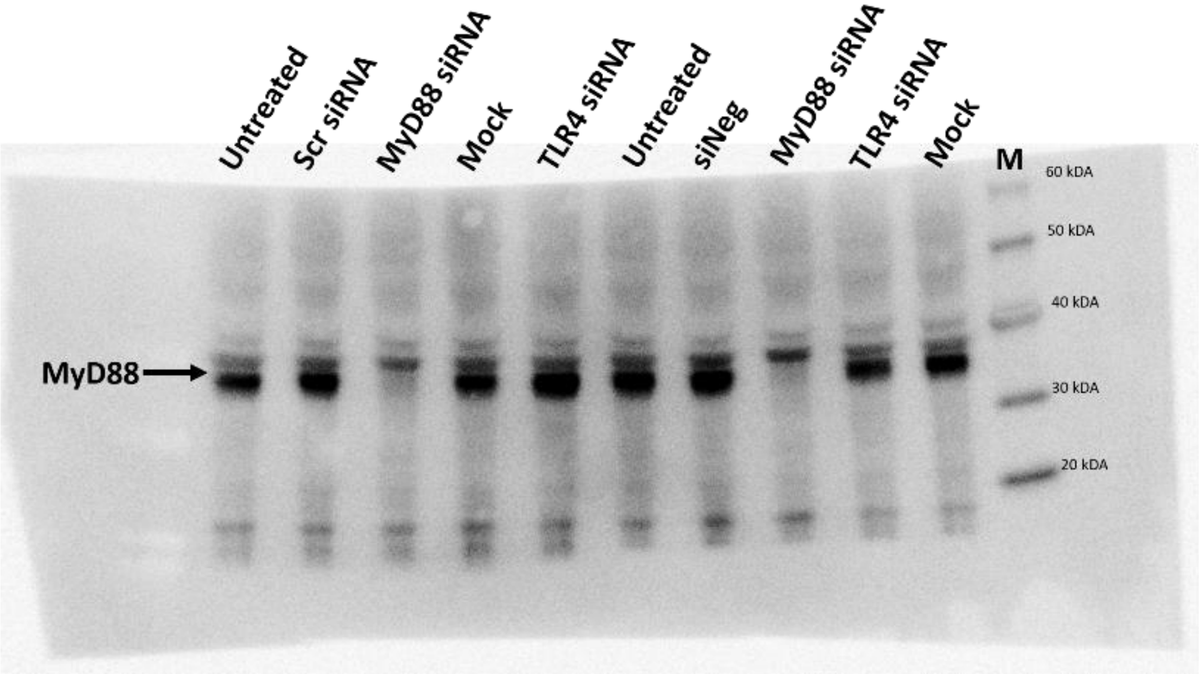

MyD88 protein expression in SKOV-3 cells following knockdown of MyD88 or TLR4. The blot was exposed for 60 seconds for optimum results. The band corresponding to MyD88 is indicated by the arrow. This blot represents the first two of four replicates.

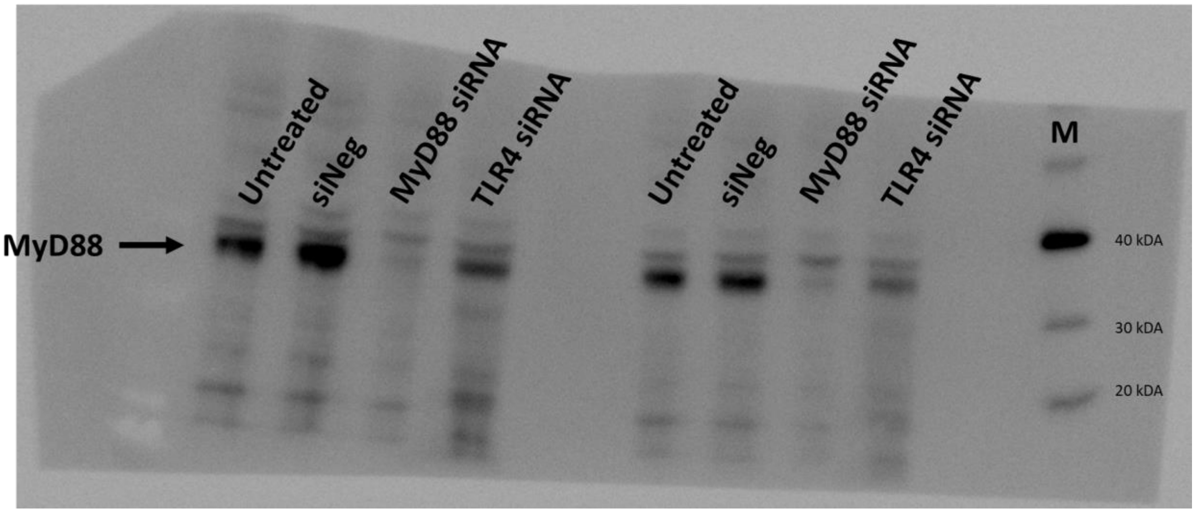

MyD88 protein expression in SKOV-3 cells following knockdown of MyD88 or TLR4. The blot was exposed for 60 seconds for optimum results. The band corresponding to MyD88 is indicated by the arrow. This blot represents the third and fourth of four replicates.

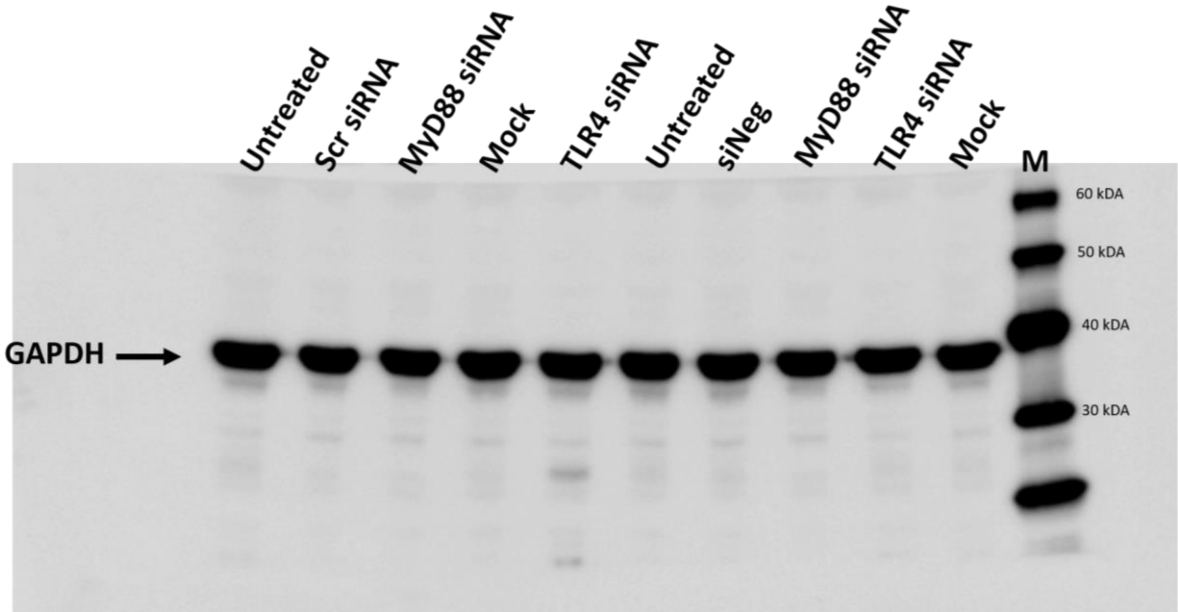

GAPDH protein expression in SKOV-3 cells following knockdown of MyD88 or TLR4. The blot was exposed for 140 seconds for optimum results. The band corresponding to GAPDH is indicated by the arrow. This blot represents the first two of four replicates.

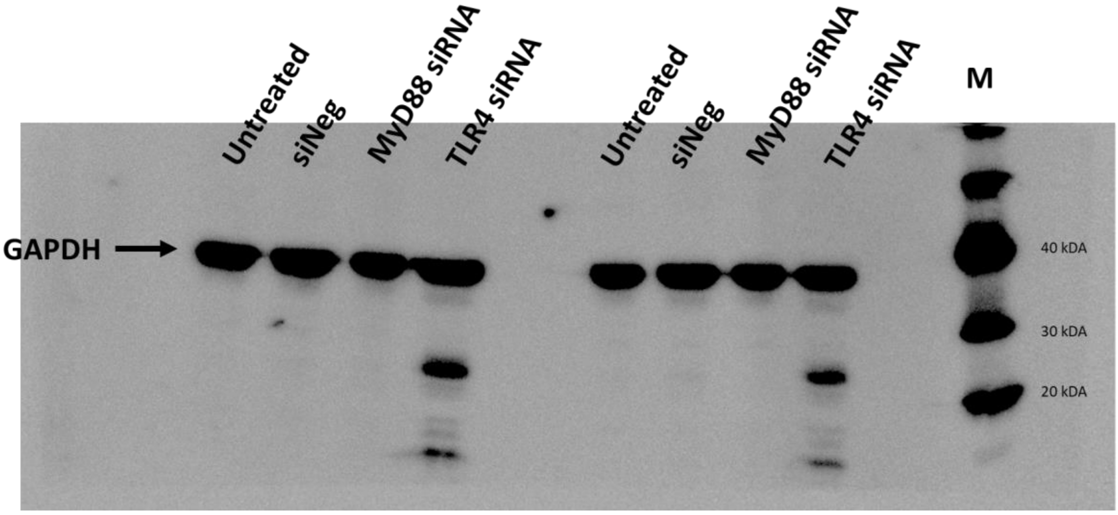

GAPDH protein expression in SKOV-3 cells following knockdown of MyD88 or TLR4. The blot was exposed for 140 seconds for optimum results. The band corresponding to GAPDH is indicated by the arrow. This blot represents the third and fourth of four replicates.

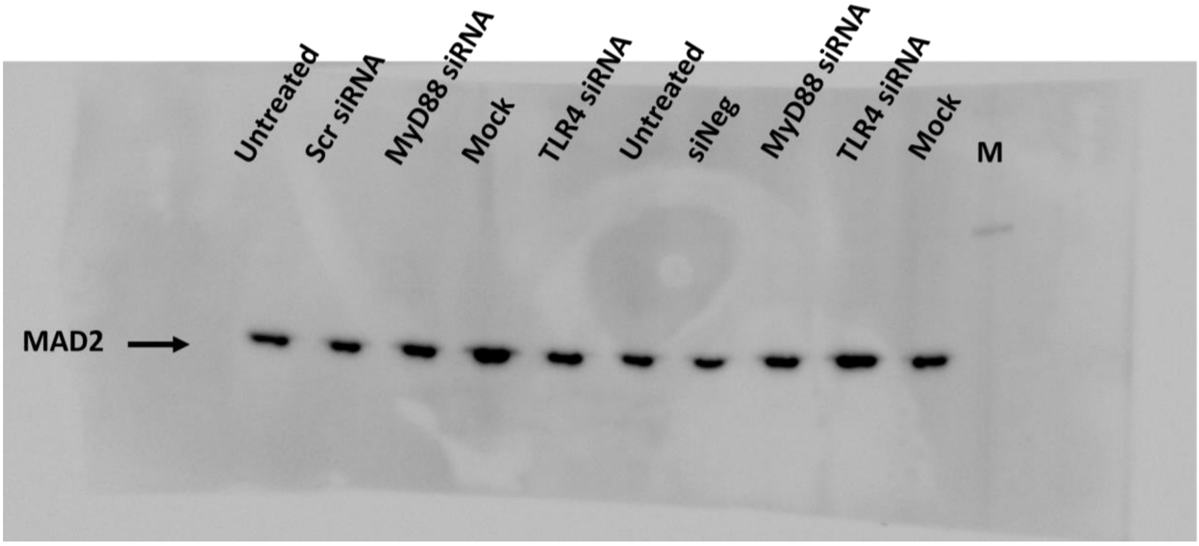

MAD2 protein expression in SKOV-3 cells following knockdown of MyD88 or TLR4. The blot was exposed for 60 seconds for optimum results. The band corresponding to MAD2 is indicated by the arrow. This blot represents the first two of four replicates.

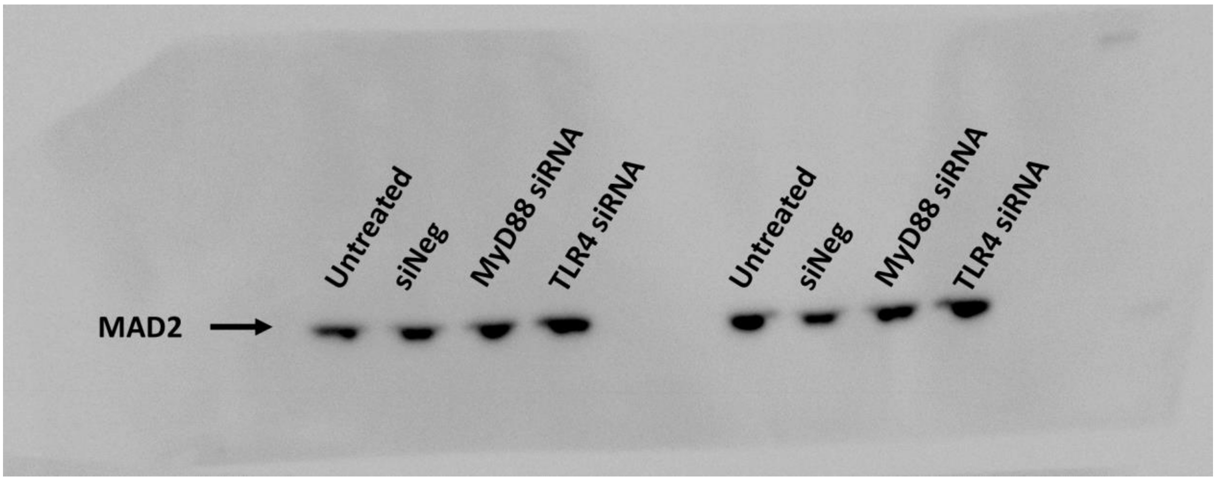

MAD2 protein expression in SKOV-3 cells following knockdown of MyD88 or TLR4. The blot was exposed for 60 seconds for optimum results. The band corresponding to MAD2 is indicated by the arrow. This blot represents the third and fourth of four replicates.

Western Blot Results Figure 1G

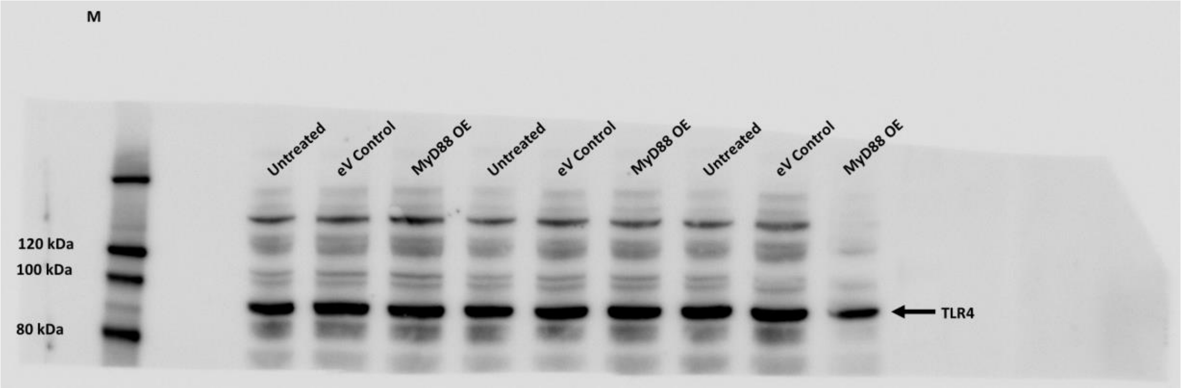

TLR4 protein expression in A2780 cells following overexpression of MyD88. The blot was exposed for 60 seconds for optimum results. The band corresponding to TLR4 is indicated by the arrow.

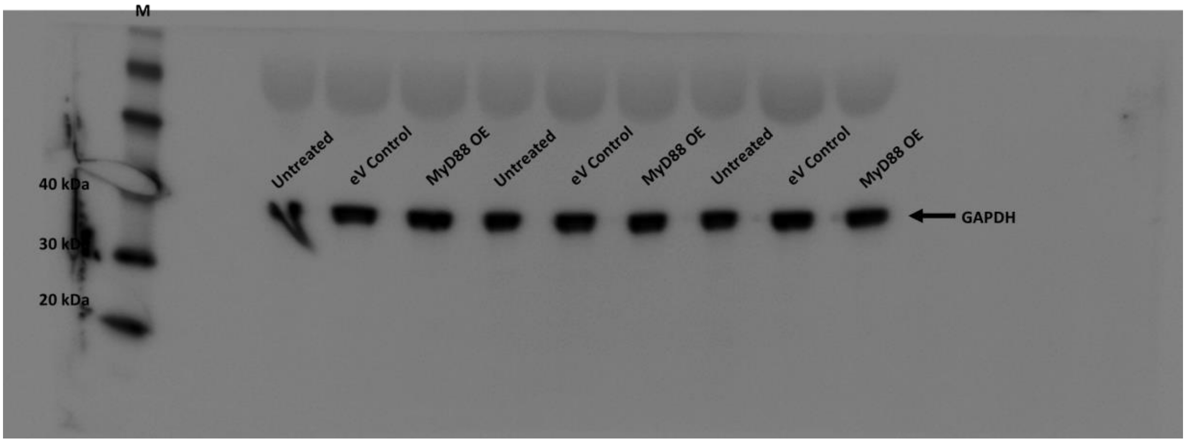

GAPDH protein expression in A2780 cells following overexpression of MyD88. The blot was exposed for 30 seconds for optimum results. The band corresponding to GAPDH is indicated by the arrow.

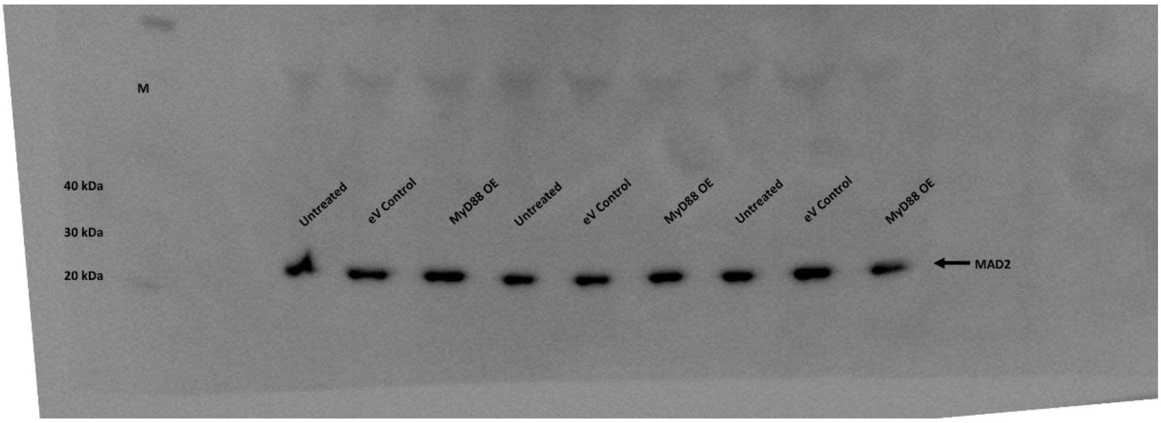

MAD2 protein expression in A2780 cells following overexpression of MyD88. The blot was exposed for 60 seconds for optimum results. The band corresponding to MAD2 is indicated by the arrow.

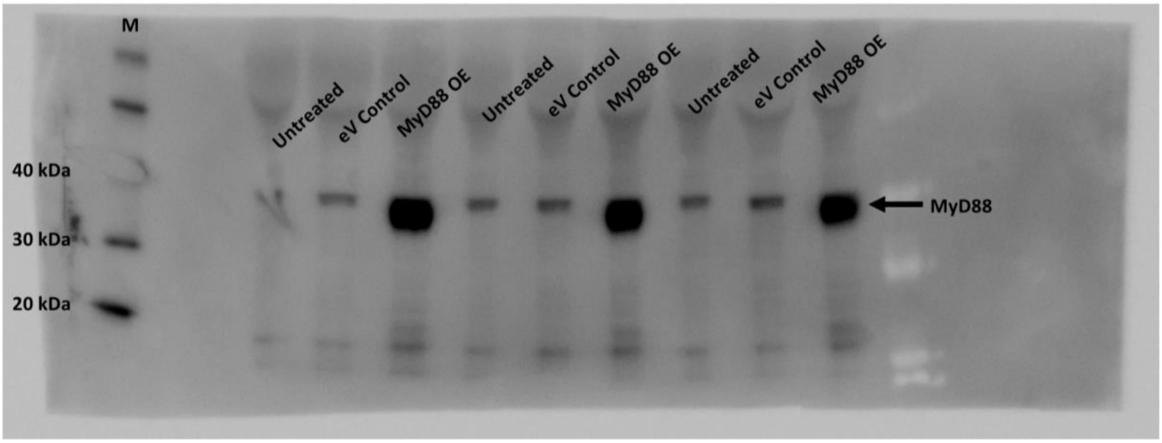

MyD88 protein expression in A2780 cells following overexpression of MyD88. The blot was exposed for 40 seconds for optimum results. The band corresponding to MyD88 is indicated by the arrow.

Western Blot Results Figure 2B

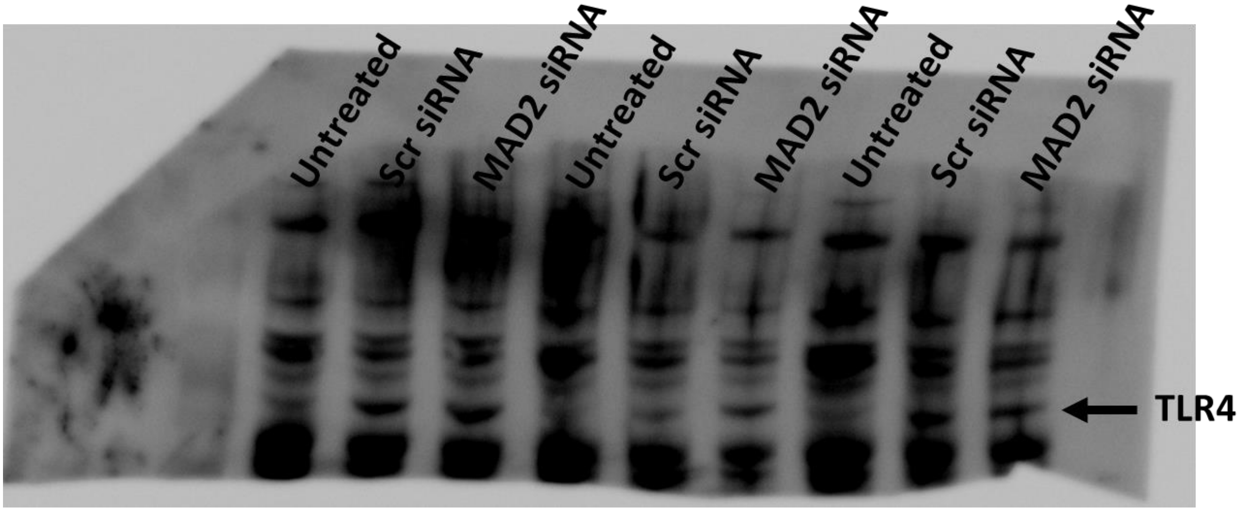

TLR4 protein expression in A2780 cells following siRNA knockdown of MAD2. The blot was exposed for 100 seconds for optimum results. The band corresponding to TLR4 is indicated by the arrow.

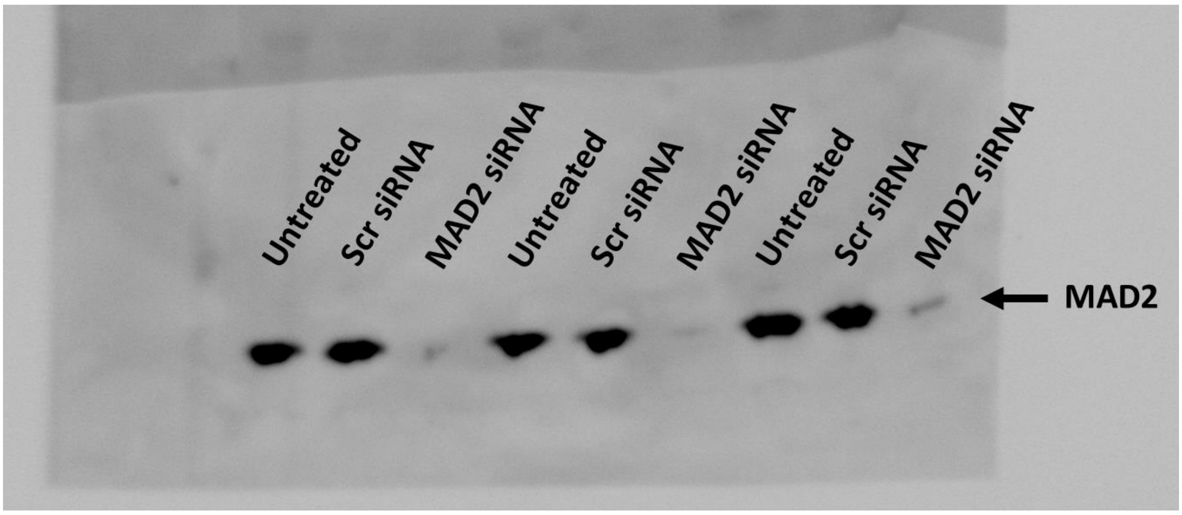

MAD2 protein expression in A2780 cells following siRNA knockdown of MAD2. The blot was exposed for 60 seconds for optimum results. The band corresponding to MAD2 is indicated by the arrow.

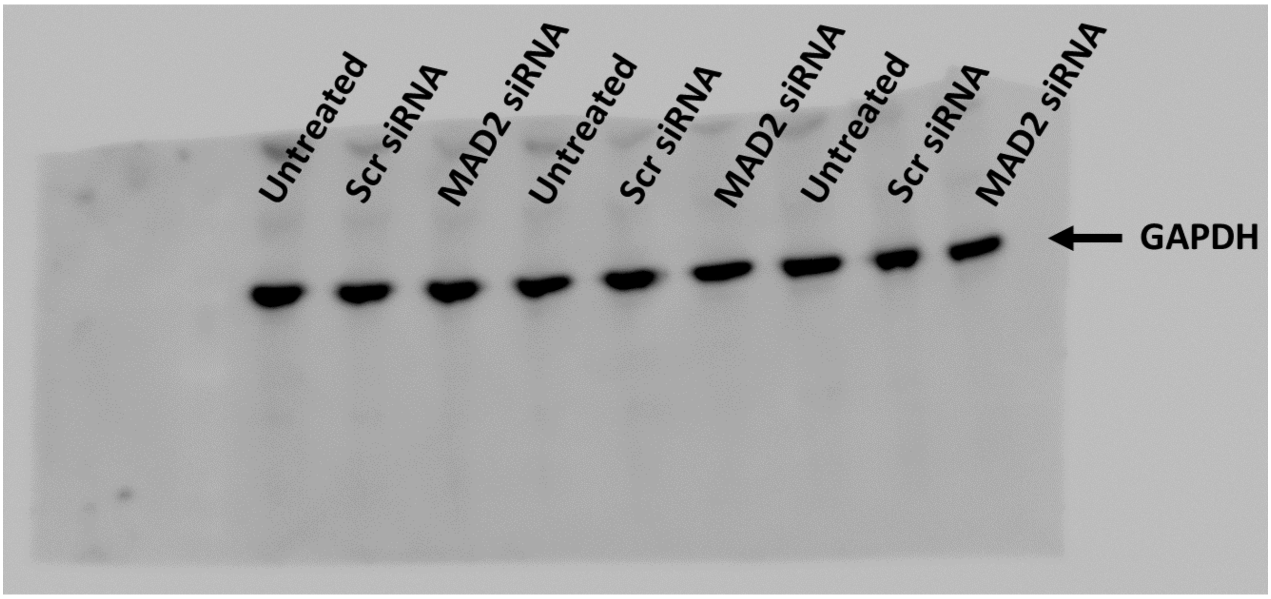

GAPDH protein expression in A2780 cells following siRNA knockdown of MAD2. The blot was exposed for 100 seconds for optimum results. The band corresponding to GAPDH is indicated by the arrow.

Western Blot Results Figure 2E

Protein lysates used in Figure 2C were obtained from SKOV-3 cells in which MAD2 was knocked down using siRNA. Membranes were probed for GAPDH, MyD88 and TLR4 and MAD2 to confirm the knockdown and to determine whether disruption of MAD2 protein expression influenced TLR4/MyD88 signalling.

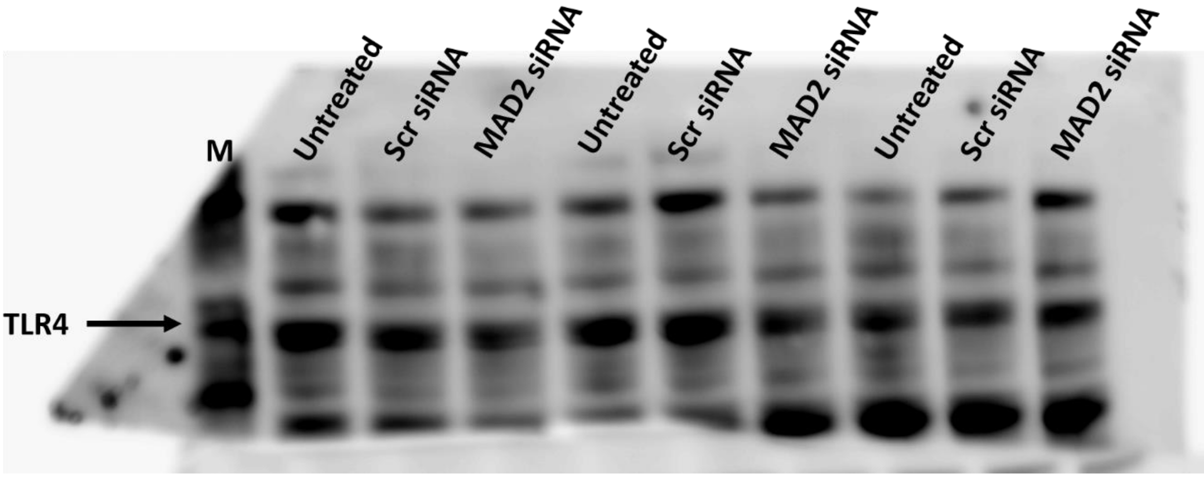

TLR4 protein expression in SKOV-3 cells following siRNA knockdown of MAD2. The blot was exposed for 150 seconds for optimum results. The band corresponding to TLR4 is indicated by the arrow.

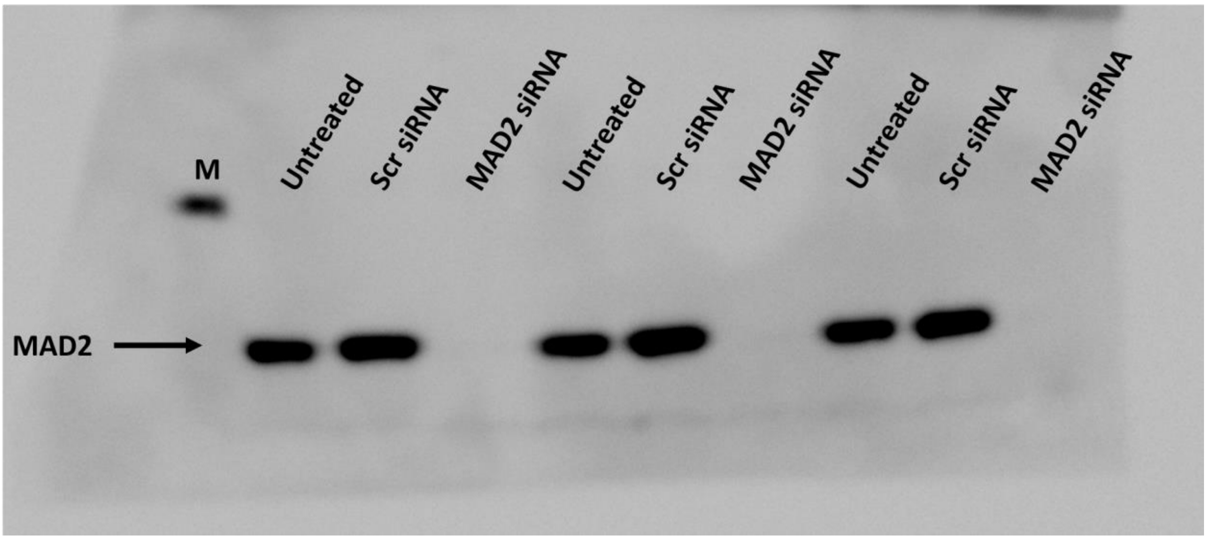

MAD2 protein expression in SKOV-3 cells following siRNA knockdown of MAD2. The blot was exposed for 100 seconds for optimum results. The band corresponding to GAPDH is indicated by the arrow.

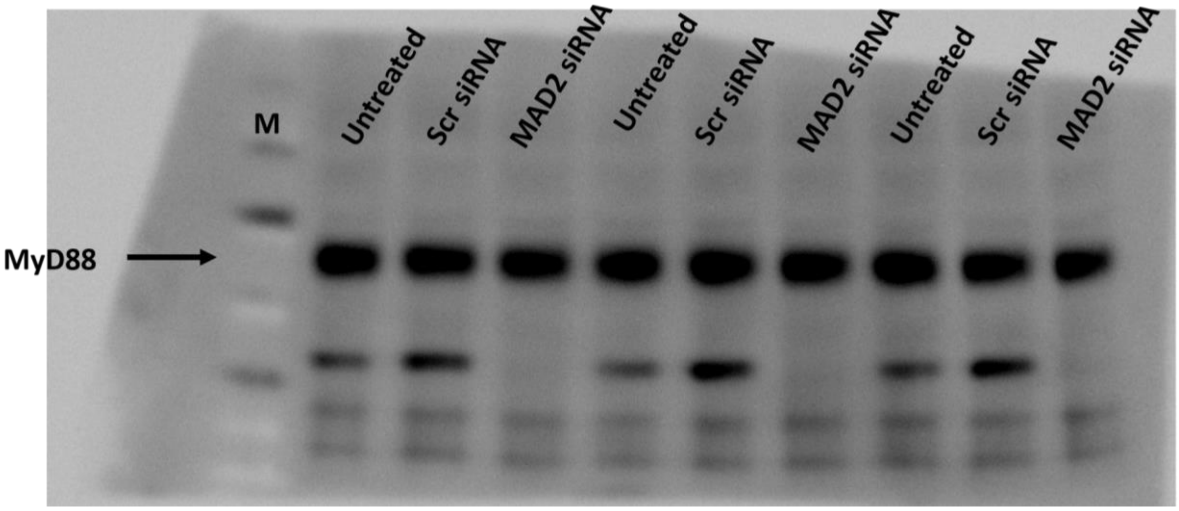

MyD88 protein expression in SKOV-3 cells following siRNA knockdown of MAD2. The blot was exposed for 160 seconds for optimum results. The band corresponding to MyD88 is indicated by the arrow.

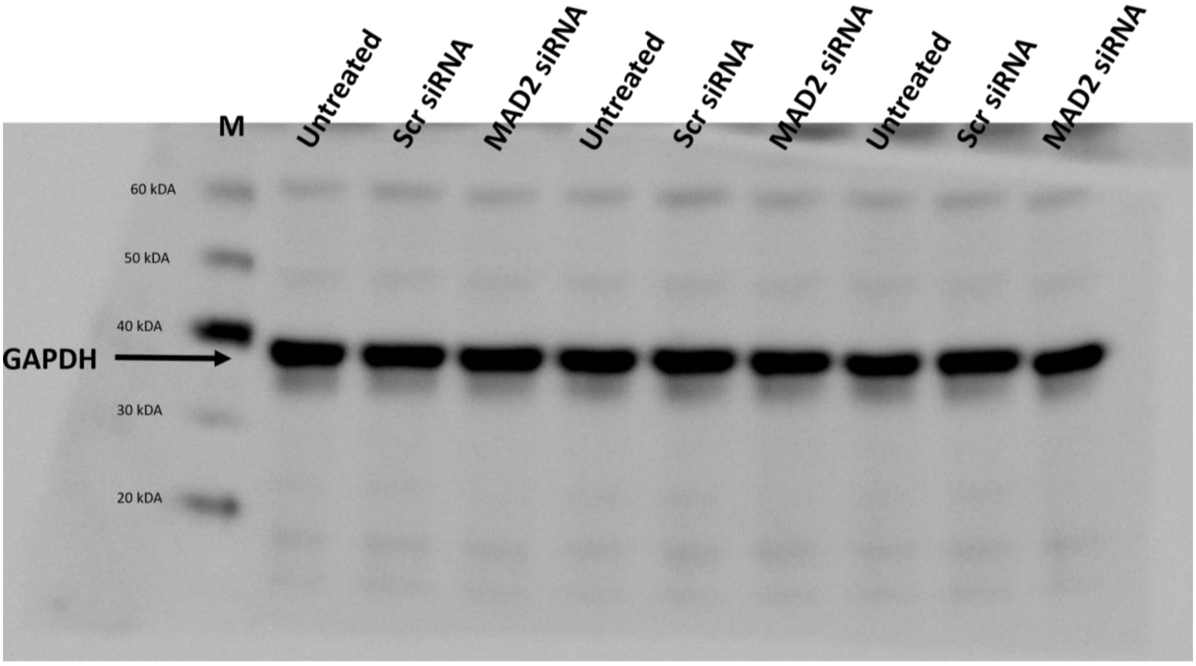

GAPDH protein expression in SKOV-3 cells following siRNA knockdown of MAD2. The blot was exposed for 80 seconds for optimum results. The band corresponding to GAPDH is indicated by the arrow.

Western Blot Results Figure 5B

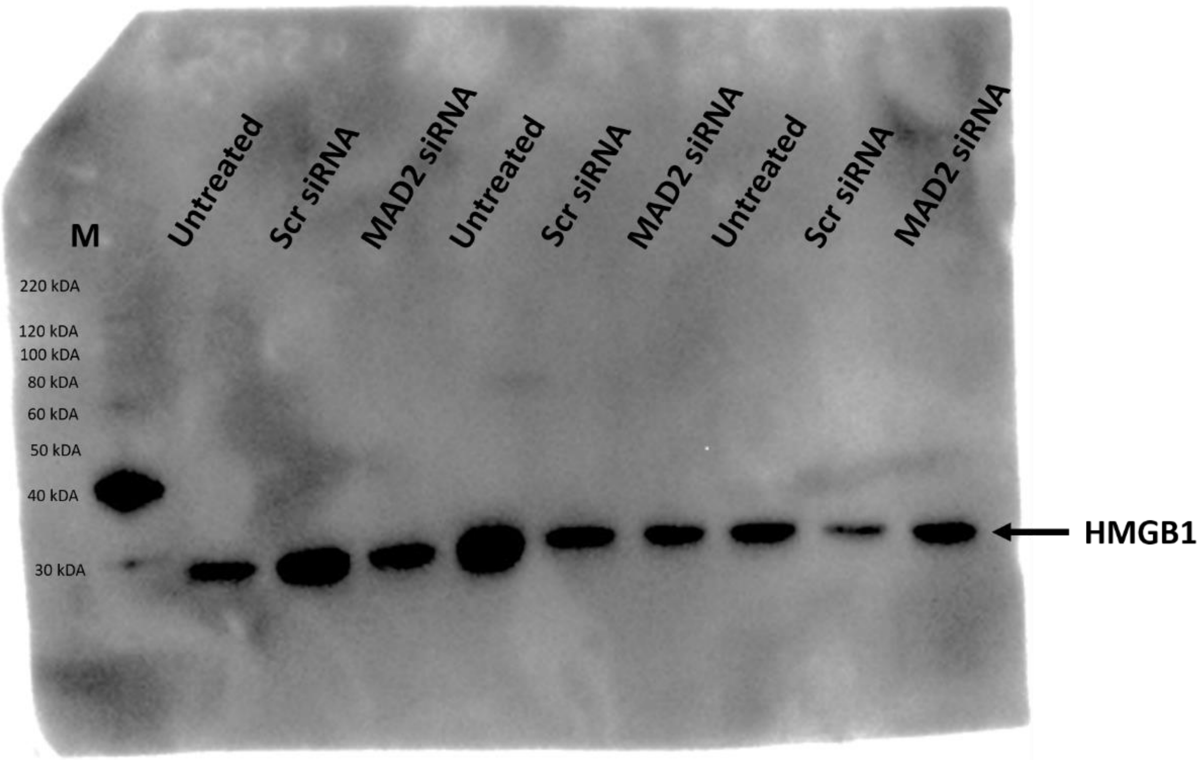

HMGB1 protein expression in A2780 cells following siRNA knockdown of MAD2. The blot was exposed for 10 seconds for optimum results. Molecular weight was confirmed using the chemiluminescence molecular weight marker (M). The band corresponding to HMGB1 is indicated by the arrow.

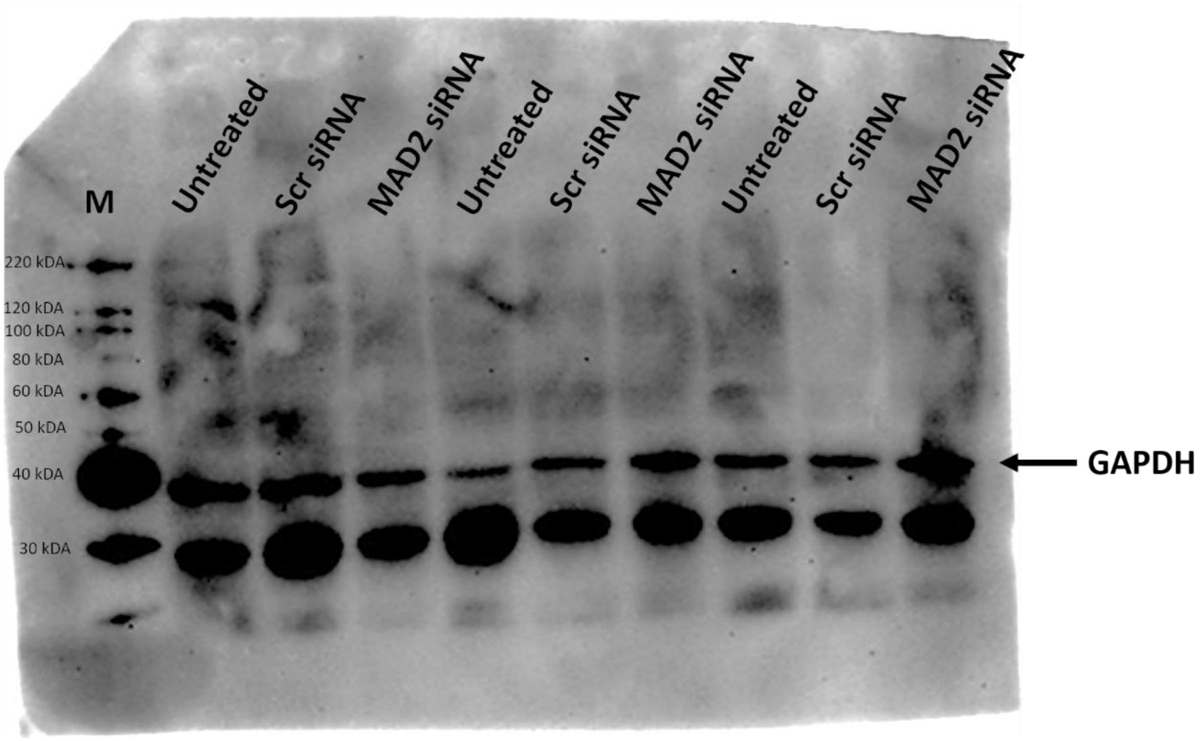

GAPDH protein expression in A2780 cells following siRNA knockdown of MAD2. The blot was exposed for 60 seconds for optimum results. Molecular weight was confirmed using the chemiluminescence molecular weight marker (M). The band corresponding to GAPDH is indicated by the arrow.

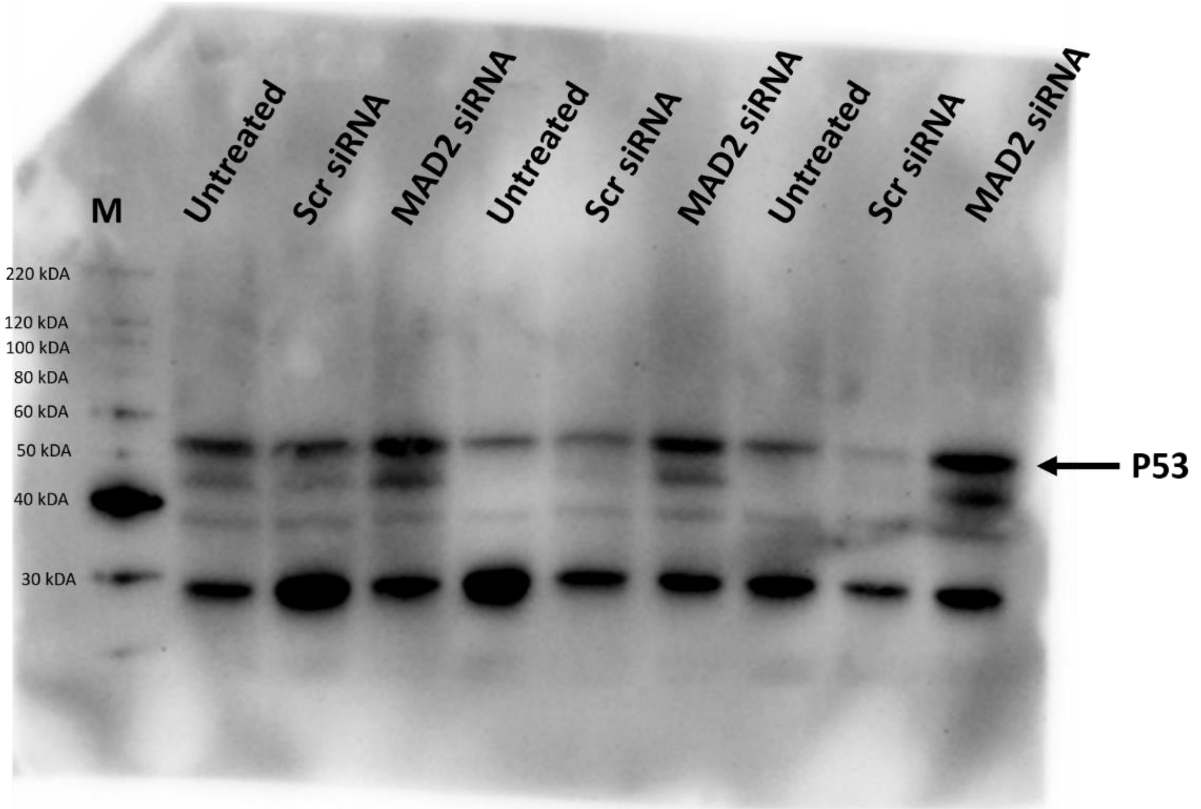

P53 protein expression in A2780 cells following siRNA knockdown of MAD2. The blot was exposed for 60 seconds for optimum results. Molecular weight was confirmed using the chemiluminescence molecular weight marker (M). The band corresponding to P53 is indicated by the arrow.

Western Blot Results Figure 5E

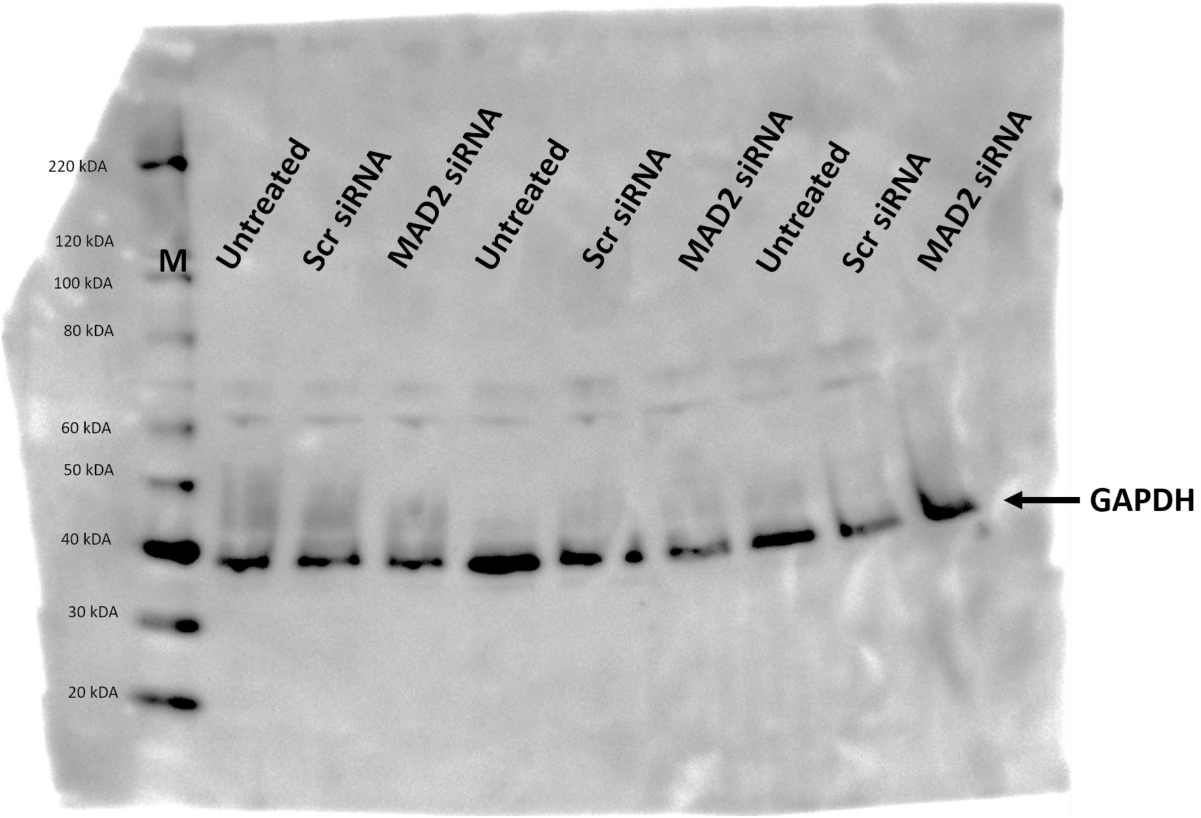

GAPDH protein expression in SKOV-3 cells following siRNA knockdown of MAD2. The blot was exposed for 60 seconds for optimum results. Molecular weight was confirmed using the chemiluminescence molecular weight marker (M). The band corresponding to GAPDH is indicated by the arrow.

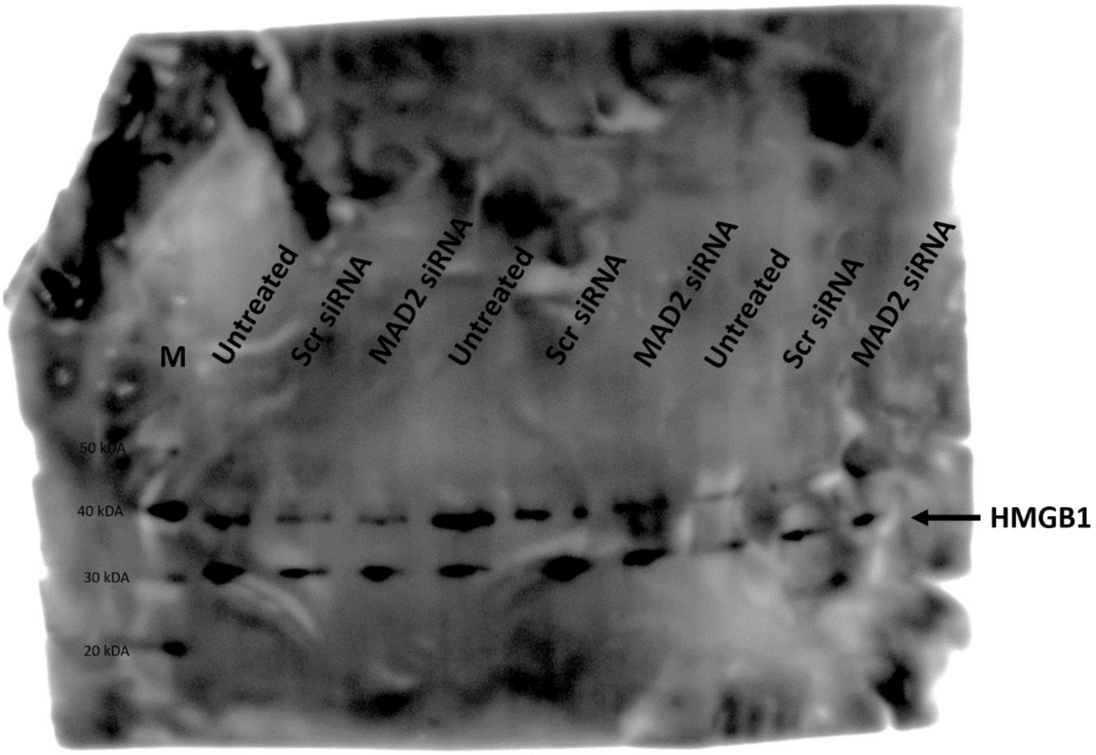

HMGB1 protein expression in SKOV-3 cells following siRNA knockdown of MAD2. The blot was exposed for 10 seconds for optimum results Molecular weight was confirmed using the chemiluminescence molecular weight marker (M). The band corresponding to HMGB1 is indicated by the arrow.
