# Supplementary material for: The role of the MAD2-TLR4-MyD88 axis in paclitaxel resistance in ovarian cancer
Source: PLoS One. 2020 Dec 28;15(12):e0243715. doi: 10.1371/journal.pone.0243715 (PMC7769460; doi:10.1371/journal.pone.0243715)
Supplement: S1 File — (DOCX) [file pone.0243715.s004.docx]

**The role of the MAD2-TLR4-MyD88 axis**

**in paclitaxel resistance in ovarian cancer**

Mark Bates PhD^1,2,3,4^*, Cathy D Spillane PhD^1,2,3^, Michael F Gallagher PhD^1,2,3^, Amanda McCann PhD^5^, Cara Martin PhD^1,2,3,6^, Gordon Blackshields PhD^2,3,6^, Helen Keegan PhD^1,2,3,6^, Luke Gubbins PhD^5^, Robert Brooks PhD^7^, Doug Brooks PhD^7^, Stavros Selemidis^8^, Sharon O’Toole PhD^1,2,3,4¶^, John J O’Leary MD, PhD^1,2,3,6¶^

^1^ Department of Histopathology, Trinity College Dublin, Dublin, Ireland

^2^ Emer Casey Molecular Pathology Research Laboratory, Coombe Women & Infants University Hospital, Dublin, Ireland

^3^ Trinity St James's Cancer Institute, Dublin, Ireland

^4^ Department of Obstetrics and Gynaecology, Trinity College Dublin, Dublin, Ireland

^5^ College of Health Sciences, University College Dublin, Belfield, Dublin, Ireland

^6^ Department of Pathology, Coombe Women & Infants University Hospital, Dublin, Ireland

^7^ School of Pharmacy and Medical Sciences, University of South Australia, Adelaide, Australia

^8^School of Health and Biomedical Sciences, Royal Melbourne Institute of Technology, Bundoora, Australia

¶Joint Senior Author

*Corresponding author

Email: [**batesm1@tcd.ie**](mailto:batesm1@tcd.ie) **(MB)**

**A2780 and SKOV3 Dose Response Curves**


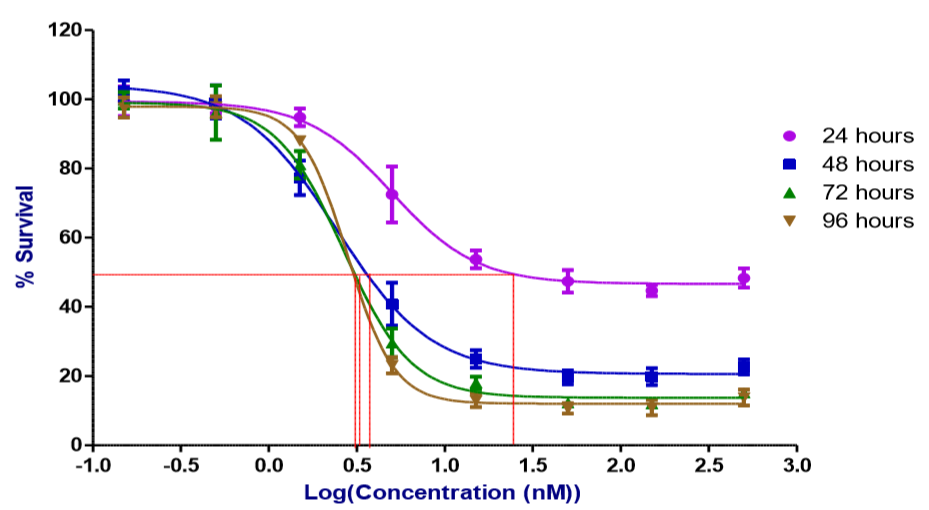


**A2780 paclitaxel dose response curve.**

AAa A2780 cells were treated with various doses of paclitaxel for 24, 48, 72 and 96 hours. Cell viability was assessed by an MTT after each time point. The IC50 value for SKOV-3 at 24,48,72 and 96 hours were 4nM, 3.1nM, 3.4nM and 2.7nM.

**
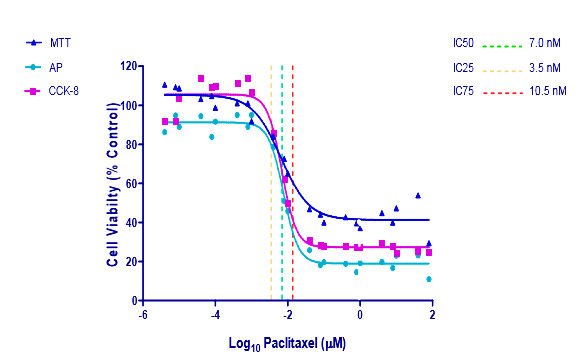
**

**SKOV-3 paclitaxel dose response curve.**

SKOV-3 cells were treated with various doses of paclitaxel for 48 hours. Cell viability was assessed by three different assays, the cell counting kit 8 (CCK-8), the (4,5-dimethylthiazol-2-yl)-2,5-diphenyltetrazolium bromide (MTT) and Alkaline phosphatase (AP) assay. The IC50 value for SKOV-3 at this timepoint was 7nM.


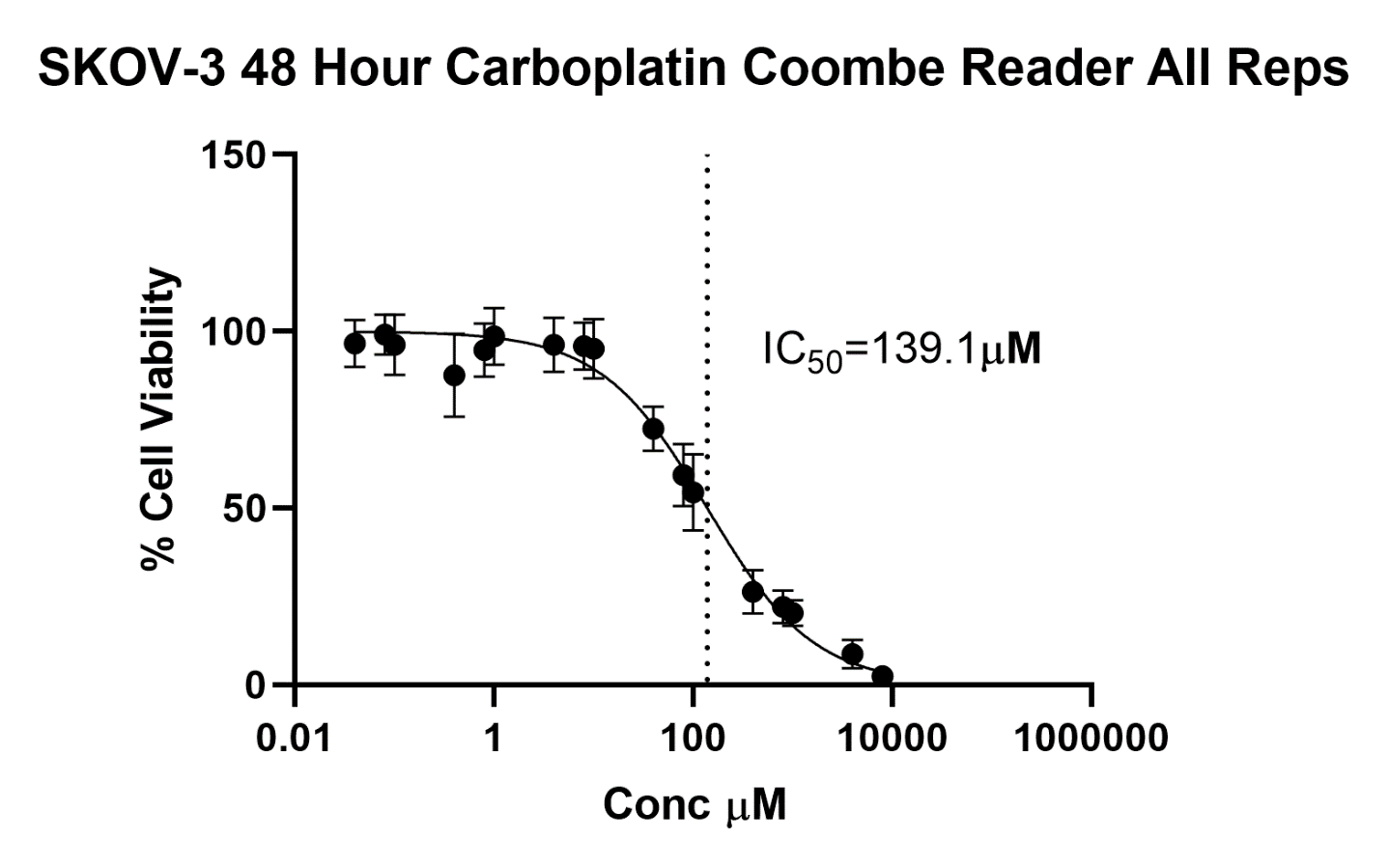


**SKOV-3 carboplatin dose response curve.**

SKOV-3 were cells treated with various doses of carboplatin for 48 hours. Cell viability was assessed using the cell counting kit 8 (CCK-8). The IC50 value for SKOV-3 at this timepoint was 139.1µM.
