# Supplementary material for: The role of the MAD2-TLR4-MyD88 axis in paclitaxel resistance in ovarian cancer
Source: PLoS One. 2020 Dec 28;15(12):e0243715. doi: 10.1371/journal.pone.0243715 (PMC7769460; doi:10.1371/journal.pone.0243715)
Supplement: S1 Table — (DOCX) [file pone.0243715.s005.docx]

**S1 Table.** **Significantly over-represented biological processes identified by the DAVID database following knockdown of TLR4 in SKOV-3 cells**. **Biological Process** – The different biological processes over-represented following knockdown of MAD2 are represented in column 1. **P-value** – the significance value of the expression change observed, a p value of ≤0.05 was set as the threshold for a significant alteration in a biological process. **Count** – The number of genes involved in a particular biological process which was found to be over-represented. **%** – Percentage of genes affected out of the total number of genes altered following knockdown of TLR4.

| **Biological Process** | **Count** | **%** | **P-Value** |
| --- | --- | --- | --- |
| Oxidoreductase activity | 3 | 0.7 | 0.0074 |
| Chromatin binding | 10 | 2.4 | 0.0078 |
| Kinase regulator activity | 7 | 1.7 | 0.0200 |
| Enzyme inhibitor activity | 13 | 3.1 | 0.0220 |
| Prenyltransferase activity | 3 | 0.7 | 0.0400 |
| Sterol desaturase activity | 2 | 0.5 | 0.0450 |
| C-5 sterol desaturase activity | 2 | 0.5 | 0.0450 |
| Protein kinase inhibitor activity | 4 | 0.9 | 0.0490 |
| Pathways in cancer | 21 | 5 | 0.0006 |
| ErbB signalling pathway | 8 | 1.9 | 0.0096 |
| Steroid biosynthesis | 4 | 0.9 | 0.0100 |
| Complement and coagulation cascades | 7 | 1.7 | 0.0110 |
| Focal adhesion | 12 | 2.8 | 0.0220 |
| ECM-receptor interaction | 7 | 1.7 | 0.0270 |
| Renal cell carcinoma | 6 | 1.4 | 0.0430 |
| Pancreatic cancer | 6 | 1.4 | 0.0470 |
| Regulation of programmed cell death | 41 | 9.7 | <0.0001 |
| Regulation of cell death | 41 | 9.7 | <0.0001 |
| Regulation of apoptosis | 40 | 9.4 | <0.0001 |
| Regulation of cell adhesion | 13 | 3.1 | 0.0001 |
| Epidermis development | 15 | 3.5 | 0.0001 |
| Wound healing | 15 | 3.5 | 0.0002 |
| Regulation of cell proliferation | 36 | 8.5 | 0.0002 |
| Ectoderm development | 15 | 3.5 | 0.0002 |
| Positive regulation of apoptosis | 23 | 5.4 | 0.0005 |
| Positive regulation of programmed cell death | 23 | 5.4 | 0.0005 |
| Positive regulation of cell death | 23 | 5.4 | 0.0005 |
| Sterol biosynthetic process | 6 | 1.4 | 0.0012 |
| Cell division | 17 | 4 | 0.0015 |
| Regulation of cell-cell adhesion | 5 | 1.2 | 0.0015 |
| Negative regulation of programmed cell death | 19 | 4.5 | 0.0018 |
| Negative regulation of cell death | 19 | 4.5 | 0.0019 |
| Mitosis | 14 | 3.3 | 0.0019 |
| Nuclear division | 14 | 3.3 | 0.0019 |
| M phase of mitotic cell cycle | 14 | 3.3 | 0.0023 |
| Transmembrane receptor protein tyrosine kinase signalling | 14 | 3.3 | 0.0023 |
| Enzyme linked receptor protein signalling pathway | 18 | 4.2 | 0.0027 |
| Organelle fission | 14 | 3.3 | 0.0027 |
| Response to wounding | 24 | 5.7 | 0.0030 |
| Negative regulation of cell adhesion | 6 | 1.4 | 0.0030 |
| Negative regulation of T cell proliferation | 5 | 1.2 | 0.0033 |
| Cell cycle phase | 20 | 4.7 | 0.0037 |
| Negative regulation of apoptosis | 18 | 4.2 | 0.0038 |
| Phosphoinositide-mediated signalling | 8 | 1.9 | 0.0043 |
