# Supplementary material for: The role of the MAD2-TLR4-MyD88 axis in paclitaxel resistance in ovarian cancer
Source: PLoS One. 2020 Dec 28;15(12):e0243715. doi: 10.1371/journal.pone.0243715 (PMC7769460; doi:10.1371/journal.pone.0243715)
Supplement: S2 Table — (DOCX) [file pone.0243715.s006.docx]

**S2 Table. Features of senescence highlighted by microarray analysis following knockdown of MAD2 in SKOV-3 cells for 72 hours. Biological Process** – The different biological processes over-represented following knockdown of MAD2 are represented in column 1. **P-value** – the significance value of the expression change observed, a p value of ≤0.05 was set as the threshold for a significant alteration in a biological process. **Count** – The number of genes involved in a particular biological process which was found to be over-represented. **%** – Percentage of genes affected out of the total number of genes altered following knockdown of MAD2.

| **Biological Processes** | **Count** | **%** | **P-Value** |
| --- | --- | --- | --- |
| Olfactory receptor activity | 12 | 5.7 | 0.0081 |
| Insulin-like growth factor binding | 3 | 1.4 | 0.0310 |
| Phospholipase activity | 4 | 1.9 | 0.0640 |
| Insulin-like growth factor I binding | 2 | 0.9 | 0.0640 |
| Transcription coactivator activity | 6 | 2.8 | 0.0880 |
| Lipase activity | 4 | 1.9 | 0.0980 |
| Nucleosome assembly | 7 | 3.3 | 0.0004 |
| Chromatin assembly | 7 | 3.3 | 0.0004 |
| Protein-DNA complex assembly | 7 | 3.3 | 0.0005 |
| Chromatin assembly or disassembly | 8 | 3.8 | 0.0006 |
| Nucleosome organization | 7 | 3.3 | 0.0006 |
| DNA packaging | 7 | 3.3 | 0.0020 |
| Sensory perception of smell | 13 | 6.2 | 0.0032 |
| Response to endogenous stimulus | 12 | 5.7 | 0.0057 |
| Sensory perception of chemical stimulus | 13 | 6.2 | 0.0073 |
| Biomineral formation | 4 | 1.9 | 0.0080 |
| Ossification | 6 | 2.8 | 0.0093 |
| Response to organic cyclic substance | 6 | 2.8 | 0.0110 |
| Bone development | 6 | 2.8 | 0.0120 |
| Response to organic substance | 16 | 7.6 | 0.0150 |
| Response to tropane | 3 | 1.4 | 0.0190 |
| Response to cocaine | 3 | 1.4 | 0.0190 |
| Collagen catabolic process | 3 | 1.4 | 0.0210 |
| Regulation of locomotion | 7 | 3.3 | 0.0210 |
| Response to steroid hormone stimulus | 7 | 3.3 | 0.0210 |
| Response to hormone stimulus | 10 | 4.7 | 0.0220 |
| Response to alkaloid | 4 | 1.9 | 0.0220 |
| Cognition | 18 | 8.5 | 0.0250 |
| Regulation of blood pressure | 5 | 2.4 | 0.0260 |
| Negative regulation of cell migration | 4 | 1.9 | 0.0260 |
| Regulation of ion transmembrane transporter activity | 3 | 1.4 | 0.0290 |
| Response to oestrogen stimulus | 5 | 2.4 | 0.0300 |
| Negative regulation of locomotion | 4 | 1.9 | 0.0310 |
| Regulation of foam cell differentiation | 3 | 1.4 | 0.0310 |
| Regulation of ion transmembrane transport | 3 | 1.4 | 0.0310 |
| Negative regulation of cell motion | 4 | 1.9 | 0.0330 |
| Multicellular organismal catabolic process | 3 | 1.4 | 0.0340 |
| Regulation of transmembrane transporter activity | 3 | 1.4 | 0.0340 |
| Negative regulation of macromolecule metabolic process | 15 | 7.1 | 0.0350 |
| Regulation of transmembrane transport | 3 | 1.4 | 0.0360 |
| Defence response to bacterium | 5 | 2.4 | 0.0370 |
| Sensory perception | 16 | 7.6 | 0.0370 |
| Negative regulation of signal transduction | 7 | 3.3 | 0.0380 |
| Collagen metabolic process | 3 | 1.4 | 0.0390 |
| Regulation of cell migration | 6 | 2.8 | 0.0410 |
| Negative regulation of smooth muscle cell migration | 2 | 0.9 | 0.0440 |
| Chromosome organization | 11 | 5.2 | 0.0450 |
| Multicellular organismal macromolecule metabolic process | 3 | 1.4 | 0.0470 |
